# Nuclear war between Israel and Iran: lethality beyond the pale

**DOI:** 10.1186/1752-1505-7-10

**Published:** 2013-05-10

**Authors:** Cham E Dallas, William C Bell, David J Stewart, Antonio Caruso, Frederick M Burkle, Jr

**Affiliations:** 1 Institute for Disaster Management, College of Public Health, 001 Barrow Hall, Athens GA 30633, USA; 2 Institute for Disaster Management, College of Public Health, 003 Barrow Hall, Athens GA 30633, USA; 3 Bartlett & West, 1200 SW Executive Drive, Topeka, KS 66615-3850, USA; 4 Institute for Disaster Management, College of Public Health, 007 Barrow Hall, Athens, GA 30633, USA; 5 Woodrow Wilson International Center for Scholars, Washington DC Harvard Humanitarian Initiative, Harvard School of Public Health, Harvard University, Cambridge, USA

**Keywords:** Nuclear war, Mass casualty, Radiation, Thermal burns, Trauma, Iran, Israel

## Abstract

**Background:**

The proliferation of nuclear technology in the politically volatile Middle East greatly increases the likelihood of a catastrophic nuclear war. It is widely accepted, while not openly declared, that Israel has nuclear weapons, and that Iran has enriched enough nuclear material to build them. The medical consequences of a nuclear exchange between Iran and Israel in the near future are envisioned, with a focus on the distribution of casualties in urban environments.

**Methods:**

Model estimates of nuclear war casualties employed ESRI's ArcGIS 9.3, blast and prompt radiation were calculated using the Defense Nuclear Agency's WE program, and fallout radiation was calculated using the Defense Threat Reduction Agency's (DTRA's) Hazard Prediction and Assessment Capability (HPAC) V404SP4, as well as custom GIS and database software applications. Further development for thermal burn casualties was based on Brode, as modified by Binninger, to calculate thermal fluence. ESRI ArcGISTM programs were used to calculate affected populations from the Oak Ridge National Laboratory's LandScanTM 2007 Global Population Dataset for areas affected by thermal, blast and radiation data.

**Results:**

Trauma, thermal burn, and radiation casualties were thus estimated on a geographic basis for three Israeli and eighteen Iranian cities. Nuclear weapon detonations in the densely populated cities of Iran and Israel will result in an unprecedented millions of numbers of dead, with millions of injured suffering without adequate medical care, a broad base of lingering mental health issues, a devastating loss of municipal infrastructure, long-term disruption of economic, educational, and other essential social activity, and a breakdown in law and order.

**Conclusions:**

This will cause a very limited medical response initially for survivors in Iran and Israel. Strategic use of surviving medical response and collaboration with international relief could be expedited by the predicted casualty distributions and locations. The consequences for health management of thermal burn and radiation patients is the worst, as burn patients require enormous resources to treat, and there will be little to no familiarity with the treatment of radiation victims. Any rational analysis of a nuclear war between Iran and Israel reveals the utterly unacceptable outcomes for either nation.

## Introduction

The proliferation of nuclear weapons in the Middle East is leading to increasing concern for their impending use in this volatile region. While Israel has been recognized (though not declared) as having nuclear weapons for decades, the rapid pace of uranium enrichment by Iran has led to the widespread conclusion that this nation will also have a stockpile of nuclear weapons in the near future. Recent repeated declarations of a desire for the annihilation of Israel by Iranian leadership, and the propensity of Israel for preempting similar intentions by others in the past, leads to the consideration that a nuclear weapon exchange is feasible in this initial time-frame in which both Israel and Iran could have substantial nuclear weapons available to use against each other. Analysis of the impact of nuclear weapon use on American cities has revealed the shocking outcome in mortality and morbidity in densely occupied urban areas. Nuclear weapons with larger than 100 Kt yields were found to generate predominantly thermal and fallout radiation casualties, able to cause burns and generate fires at distances considerably greater than building damage, and to spread lethal levels of fallout for many kilometers, causing radiation casualties many kilometers downwind from detonation [1,2]. The key factors in the dramatic differences in impact of an Iranian/Israeli nuclear exchange will be the lower fission yields, numbers of weapons, and less accurate targeting of the Iranian forces relative to the Israeli nuclear capabilities.

In a detailed analysis of Iranian and Israeli nuclear capability by the Center for Strategic and International Studies [3], it was assumed that Israel’s hypothetical nuclear forces would entail at least 200 boosted and fusion weapons, with yields from 20 Kt to 1 megaton. Israel’s delivery capability includes 100 Jericho 1 and 2 missiles, some long range Jericho-3 missiles, nuclear-armed cruise missiles, nuclear-armed submarines and advanced aircraft with high resolution satellite precision targeting. In the near future, Iran will be predicted to have approximately 10–20 nuclear weapons, mostly fission devices, in the range of 15–30 Kt. Iranian delivery of these weapons will involve at least 100 Shahab 3 missiles (1,300 Km range), several hundred smaller Shahab missiles (300–500 Km range), some solid fuel Sejjil-2 missiles (2000 km range), Soviet and U.S. aircraft, and reverse engineered cruise missiles, with limited satellite targeting. While the recent TOR-M1 and possible S-300 acquisition of Russian defensive systems has and could further improve Iranian anti-aircraft capability, Israeli missile and aircraft superiority should be decisive in ensuring the accurate delivery of Israel’s nuclear weapons to Iranian targets.

Of the population of 7.2 million in Israel [4], the two primary targets for Iranian attack are likely to be Tel Aviv (population 391,000, metropolitan area 1.6 to 3.0 million) and Haifa. (population 270,000). For this analysis, Jerusalem is not included in targeting due to the high Muslim population (32%) and its very significant religious importance to Islam, Judaism, and Christianity. Tel Aviv is on a flat, open coastal plain, with a high population density (7,500/km2) that presents the most likely nuclear weapon target. Haifa is also on the coast, but with a range of inland hills with a “hill and valley” effect that can affect the nuclear weapons impact. A key factor for Israel is the anti-missile and anti-aircraft defenses that would be likely to limit airborne delivery of Iranian nuclear weapons into the nation, and its efficient intelligence network for the prevention of smuggled nuclear devices.

In consideration of the impact of nuclear attack within the much larger Iranian population of 69 million [4], the distinctive vulnerability of Iranian cities in general is illustrated by the capital, Tehran, with 12.6 million in the greater metropolitan area. The distinctive basin topology, reflective characteristics of the surrounding mountains, building construction, and densely packed population makes Tehran an environment strikingly conducive to very high mortality following a nuclear attack. In addition, 50% of Iran’s industry, 30% of the nation’s public sector workforce, and most of their higher education (50 colleges and universities) are clustered in this one city. Many of these characteristics are demonstrated in other Iranian cities, particularly the lack of urban sprawl that concentrates the population density, which greatly multiplies vulnerability to nuclear attack.

The medical impact of nuclear war in Iran and Israel is presented, with emphasis on the distribution of casualties in the categories of thermal burns, radiation illness, and trauma injuries, as well as short-term mortality. To the extent feasible, political, economic, and military factors are incorporated in assumptions on the size and extent of a likely nuclear exchange between these two nations over the next decade. The staggering impact of nuclear war is the key message, with an emphasis on immediate rather than long-term medical consequences.

## Data and methods

### Study area and size of weapon

Three cities and one tactical target in Israel and eighteen cities in Iran were selected for this study of the geographic distribution, quantity, and injury category definitions of nuclear weapon detonations on urban populations. For Israel, a 15 Kt nuclear weapon was simulated; for Iran five sizes of nuclear weapons of 15, 50, 100, 250 and 500 Kt were employed. A fission fraction of 1 was assumed for the 15 Kt devices and 0.8 for the 100, 250 and 500 Kt devices. The 15, 50, 100, 250 and 500 Kt weapons were detonated at 35 meters (m), 55 m, 70 m, 95 m and 120 m heights respectively with visibilities in all cases of 20,000 m. Lowering the height of the burst for a given yield causes greater thermal effects which are somewhat offset by lower downwind fallout radiation amounts [5].

### Affected population

Daytime population estimates (in interpolated 3 arc-second grid format) were derived from Oak Ridge National Laboratory’s LandScanTM 2007 Global Population Dataset [6]. ESRI’s ArcGISTM software [7] was used to create circular buffers around the detonation point(s) for each city representing regions where the population would be exposed to blast and thermal effects of greatest interest. Fallout radiation isolines were calculated using DTRA’s HPAC v4.04SP4 [8], with “urban effects” turned on. The population grid for each city was converted to polygon data (projected to UTM-WGS84 coordinates) and the population density of each grid cell calculated. For each city scenario the circular zones of interest, HPAC plume radiation isolines, and the population grid were overlaid. Rough estimates of city population sizes were made by sketching the outlines of population clusters (based on visual inspection of the LandScanTM 2007 data [6]) and summing all interior cells. The affected population for each unique combination of blast, thermal effects and fallout zone was tabulated and casualties calculated according to values listed in Table 1. Where populations are in zones affected by more than one effect, the overall survivorship is calculated as the product of the survivorship for each effect.

**Table 1:** Rates used to calculate casualties due to prompt radiation, blast, and thermal effects

**Prompt radiation/Blast/Thermal effects**	**Fatality rate**	**Survivor injury rate**
Prompt 600 rad*	50%	99%
Prompt 300 rad*	2%	99%
12 psi	85%	100%
10 psi	85%	100%
8.1 psi	50%	85%
7.1 psi	10%	70%
4.9 psi	5%	35%
3.8 psi	2.5%	25%**
3 psi	0	5%**
2 psi	0	2%**
1 psi	0	0**
0.6 psi	0	0***
90% Mass Fire	90%	100%
50% Mass Fire	50%	95%
Eden Mass Fire	40%	50%
10% Mass Fire	10%	10%
3rd-degree burns	5%	10%
2nd-degree burns	3%	12%
1st-degree burns	0%	15%

*A 50% protection factor was applied for prompt radiation bringing the actual exposures to 300 and 150 rads, respectively.

** An additional 5% injury rate is assumed to be caused by flying glass.

***An additional 2% injury rate is assumed to be caused by flying glass.

### Weather and climate data

Weather and climate have significant effects on the impacts of nuclear detonations. Wind strength and direction greatly affect the direction, shape and size of the resultant fallout cloud. We chose the median three dimensional climates from thirty years of data for a typical Mid-September day for our models. The median monthly days are computed from data supplied with the DTRA’s HPAC [8] program that computes radiation fallout from nuclear detonations.

### Effects of a nuclear detonation

The energy from a nuclear weapon is dissipated in four main ways: thermal radiation 30-50%; fallout radiation 5-10%; blast 40-60%; ionizing radiation 5%, depending upon the design of the weapon and the detonation environment.

Thermal energy (fluence) is typically measured in calories per square centimeter (cal/cm2). Larger weapon yields increase the intensity and range of thermal effects greatly [9]. Thermal energy travels directly from the fireball unless scattered or absorbed. At thermal fluencies above 10cals/cm2 large fires can start in urban areas [10] although there is much debate about the level needed for mass fires [11-13]. When detonations result in a fireball completely below cloud level, the thermal effect can double or in extreme circumstances it can quintuple [14]. Consequently the estimates of casualties in our scenarios could be very conservative. Clouds above the fireball produce multiple reflection paths resulting in more omni-directional thermal radiation, which produces fewer radiation “shadows” from buildings. This increases burn casualties and amplifies fire ignition probabilities. Even a few large clouds in the sky, supplemented by strong thermal winds and blast damage, could greatly increase the probability of local fires starting and subsequently spreading. Indeed, the intensity of fire damage can vary greatly, such as the lack of a firestorm in the second atomic bomb at Nagasaki owing to terrain features. It is a point in fact that incendiary bombing in the Second World War in Germany at Dresden and Hamburg actually created far greater fire damage in terms of percent fatalities in population at risk versus fire severity. This was due to the fact that incendiary bombs lengthen the time of exposure to the inflammatory material relative to the brief, high intensity flash as occurred in the atomic bomb explosions at Hiroshima and Nagasaki.

Prompt, or ionizing radiation, occurs immediately after the detonation and fatal doses typically occur out to about 1,500 m for 15 Kt devices and about 2500 m for 500 Kt devices dropping off rapidly with increasing radii from the epicenter. These distances are within the mass fire zone for both of these detonations.

Fallout radiation causes a conical shaped plume that is blown downwind from ground zero. Dispersion is greatly affected by turbulence in the atmosphere which in turn mainly depends upon the topography, land use, vertical wind and temperature structure.

Blast effects cause extensive damage to buildings in cities. Blast produces shock waves which increase air pressure as they propagate from the blast center, causing buildings to collapse, and glass to shatter. High, blast associated winds can knock objects down, such as people or trees. Three to four pounds per square inch (psi) overpressure is usually enough to destroy most residential buildings and many people are either dead or injured from building collapse, being blown into objects, or hit by flying debris. Blast injury estimates vary greatly.

OTA [15] state that populations experiencing between 2psi to 5psi experience 45% of all injuries and 5% of all deaths, while 25% of the population from 1 psi to 2psi are injured, mainly by flying glass and debris. We take a conservative view of injuries due to blast alone, assuming 5-7% at 1-2psi, but overall injury rates must include overlapping blast, fire, burn, and, where appropriate, fallout and prompt radiation injuries. The 1, 2, and 3 psi levels are generally used designators for construction impact, while our use of 3.8, 4.9, 7.1, and 8.1 psi were based upon National Planning Scenario levels of 10 and 50% casualty and 10 and 50% fatality levels, respectively. Our overall injury rates are generally higher than the National Planning Scenarios estimates [16] due to our higher estimates for thermal and mass fires injuries. Table 2 illustrates the relationship between blast and thermal impacts for different sized weapons. For a 100 Kt weapon, the 3.8psi blast contour is close in radius to the Fires Highly Probable (29 cals/cm2) contour, meaning people are exposed to fire, burns and blast risks here. Additionally they may experience fallout radiation if they are in the downwind section and under the radiation plume. Further discussion of the range of casualties from 10 kt terrorist weapon in New York (Manhattan) can be seen from an analysis by Harney [17] in which traditional air burst and contrasts it with casualty effects from a surface burst. This provides a helpful information link for those interested in the rationale of the methods employed for casualty distribution calculation. It is interesting to note that the 10 kt surface burst casualty estimates in his Table three by Harney [17] for New York are much higher than the relative estimates in our typical Middle Eastern cities, indicating the method employed in this paper is not overestimating casualties relative to the available other published methodology in the literature.

**Table 2:** Level of thermal fluence (calories/cm2) and corresponding overpressure (psi) for different sized nuclear weapons

**Weapon size, Height of Burst (HOB) Fission fraction (FF)**	**15 Kt 35 m; 1.0**		**50 Kt 55 m 0.8**		**100 Kt 70 m 0.8**		**250 Kt 95 m 0.8**		**500 Kt 120 m 0.8**	
**Thermal Effect**	**Fluence**	**psi**	**Fluence**	**Psi**	**Fluence**	**psi**	**Fluence**	**psi**	**Fluence**	**psi**
Fires Highly Probable (Binninger)	23	4.6	26	4.1	29	3.8	32	3.6	35	3.6
Fires Probable (Binninger)	12	2.8	13	2.5	15	2.4	16	2.4	18	2.4
Mass Fires Likely (Eden/Postol)	10	2.5	11	2.3	12	2.2	14	2.2	15	2.2
3rd Degree Burns (50% chance)	7.4	2.1	8.1	1.9	8.5	1.8	8.9	1.7	9.3	1.8
2nd Degree Burns (50% chance)	4.8	1.2	5.2	1.3	5.5	1.4	5.8	1.4	6.0	1.4
1st Degree Burns (50% chance)	2.4	0.8	2.6	0.9	2.7	1.0	2.9	1.0	3.0	1.1

### Limitations and sources of uncertainty in the models

We used the work of Binninger [12] based on Brode's earlier work [11] to calculate thermal fluence, DTRA’s HPAC V404SP4 [8] with urban effects turned on for radiation, and the Defense Nuclear Agency’s WE program [18] for blast. With any such models there are many sources of uncertainty in the input parameters and limitations in the models themselves, given the complex city center landscape with a multitude of different construction standards and architectures. A discussion of the models and their limitations follows.

### Thermal effects – Mass fires and burns

The thermal impacts of a nuclear explosion are always large, scaling faster than blast with larger weapons, since thermal radiation decays as the inverse square while blast decays as the inverse cube of distance from the detonation point. Beginning in the 1970’s, Brode worked on fire damage, fire spread and fire modeling [11]. Eden [10] also discusses fire modeling and spread, and examines the Nuclear Weapon Fire Start Model [13]. Starting around 2000, DTRA funded fire prediction modeling to facilitate the incorporation of models within a modern computer modeling package such as HPAC [8] and we have used the underlying fire start theory from this work in our calculations. We follow Binninger's [12] values for urban thermal ignition based on data values from the Nevada test site. It should be noted, however, that the dry desert air in this scenario was more likely to be conducive to ignition and flame sustainment than the more humid air likely to often be expected in coastal cities.

The fires possible level was taken from Eden. Her belief is that 10 cal/cm2 is a good first estimate of the range out to which a mass fire could be expected in a city attack such as with a Nagasaki/Hiroshima sized weapon [10]. This would correspond to around 15 cal/cm2 for a 500 Kt detonation after application of the relevant thermal fluence equation

$$Q=W*C_{\mathrm{atm}}*S_{\mathrm{cref}}*T_{\mathrm{frac}}*\frac{\left(1+\frac{\varphi }{v}r\right)\;e^{\left(\frac{-\mathrm{\delta r}}{v}\right)}}{4\pi r^{2}}$$

Where

- Q is total thermal fluence in cal/cm2

- W = weapon yield in Kt

- r = straight-line slant distance to center of blast in meters

- v = visibility in Km

- φ = air scattering factor

- δ = clear air absorption factor

- Catm = Cloud attenuation factor

- Scref = Cloud-snow enhancement factor

- Tfrac = Thermal fraction.

Thermal fluence scales according to **Q**_**1**_**/Q**_**2**_ **= (W**_**1**_**/W**_**2**_**)0.1**2 where W_1_ and W_2_ are the sizes of the two detonations in kilotons and Q_1 _and Q_2_ are the respective thermal fluencies [10].

Eden based her analysis upon data collected from Hiroshima and believes the 20 cal/cm2 quoted by some analysts necessary for mass fires in a large city is unnecessarily conservative as common fuels are ignitable at 3 cal/cm2. Postol [19] also supports the 10 cal/cm2 mass fire level while Daugherty’s [20] 12,000 mete radius for a one megaton airburst corresponds to a thermal fluence of 9 cal/cm2 under similar atmospheric conditions. In the National Planning Scenarios [16], a lower degree of thermal impact was employed, which resulted in a somewhat less drastic outcome in the number of thermal burn casualties relative to that presented in this publication and in Harney’s analysis [17].

The Mass Fires Probable and Highly Probable values in Table 2 roughly correspond to Binninger [12] values applicable to both single family frame housing and multistory steel/concrete structures. The 500 Kt fire values are 1.5 times higher than the 15 Kt detonations according to the thermal fluence values in the Fires Highly Probable category corresponding to just over a 90% probability of ignition, while the Fires Probable level corresponds to slightly over a 50% probability of ignition. The Mass Fires Possible category from Eden [10] and Postol [19] would correspond to a probability of ignition on the Binninger [12] scale of somewhere between 10% and 50%. With a reflective cloud layer above the fireball, radiation can double, so a 10% fire ignition probability become 50%, while a 50% probability becomes 90% due to the fire ignition probability distribution. This results in the three categories of thermal burn injuries, including first degree burns which are primarily confined to the epidermis, second degree burns which extend to some extent into the underlying dermis, and the third degree burns (or full thickness burn) which involves the entire dermis.

Thermal fluences necessary for the first, second and third degree burn levels were taken from Fig. 12.65 of Glasstone & Dolan [9].

The number of uncertainties in a complex environment such as that of a modern city immediately after a nuclear explosion remains high and active debate continues about what constitutes sufficient thermal fluence for mass fires.

### Blast effects

Blast was calculated for psi values that generally followed the National Planning Scenarios [16], (Table 1) using the Defense Nuclear Agency’s WE program [18], and for distances corresponding to significant thermal events (Table 2). This results in the various categories of trauma related injuries, including primary injuries (such as tympanic membrane destruction in the ears due to the overpressure wave), secondary injuries (such as eye injuries and cuts on exposed limbs from wind-blown glass and other debris), tertiary injuries (trauma injuries resulting from the actual impact of a flying human body against structures, or from the tumbling of the body), and quaternary injuries (severe trauma resulting from building collapse).

### Fallout radiation effects

The fallout radiation was partitioned into 13 classes of free-in-air radiation doses (5–25; 25–75; 75–150; 150–300; 300–380; 380–450; 450–530; 530–680; 680–900; 900–1360; 1360–2120; 2120–4000; >4000 rads) generally following Anno’s [21] dose ranges and associated pathophysiological effects for acute radiation exposure and not the more conservative Lawrence Livermore National Laboratory’s (ES&H Manual Document 22.6 Exposure to Radiation in an Emergency) [22]. The comparison is available in Table 3. Actual radiation exposure will be affected by a myriad of factors which can result in various protection factors being applied to some of the population. Blast damage to buildings and fear of building collapse from previous and/or future detonations can cause people to run into the open and expose themselves to additional fallout radiation, particularly in earthquake sensitized populations, as in much of Iran.

**Table 3:** Impact of radiation on casualties

**Dose range (rads) Baum**	**Dose range (rads)LLNL**	**Biological effect**	**Survival**
75-150	25-100	Slight decrease in blood count; minor radiation sickness	Virtually certain
150-300	100-200	Symptoms of bone marrow damage; moderate radiation sickness	Probable (>90%)
300-530	200-300	Moderate to severe bone marrow damage; serious radiation sickness	Possible – Lower third of range LD5/60
			Middle third of range LD10/60
			Top third of range LD50/60
530-830	350-550	Severe bone marrow damage; extreme radiation sickness	Death within 24–42 days
			Bottom half of range LD90/60
			Top half LD99/60
>830	>550		Death in 2–21 days

### Limitations

Limitations of this study are inclusive to the error analysis of the Hazard Prediction and Assessment Capability (HPAC) which was developed by the Defense Threat Reduction Agency to accurately predict the release, dispersal and effects of Nuclear, Biological and Chemical hazards in support of decision makers responding to an accident or event. Such software was developed over a number of years using standard validation and verification (V&V) processes [23-25]. Table 1.1 on pages 4–6 of reference 23 summarizes the V&V activities for several versions of HPAC. According to DOD document 5000.59 [26], validation is “the process of determining the degree to which a model is an accurate representation of the real world from the perspective of the intended uses of the model”. HPAC predictions (from a simulation) were compared from observations (from real world nuclear test data) and it was felt that they met the criteria of “building the right thing” and “building the thing right” [23]. A complete error analysis is presented in Bradley et al. [23].

The HPAC model described above calculated blast and radiation consequences of a nuclear detonation well but did not adequately cover the thermal components and their consequences given HPAC’s original military focus. The thermal components of the model used in this study were developed from the theory discussed in white papers submitted by Binninger et al. to the Defense Threat Destruction Agency in 2003 [27] and 2004 [28]. The results from this model were compared to experimental field data from previous tests [29] and the simulated data matched the experimental data. Despite this, uncertainties are inherent in this methodology.

**Figure 1:**
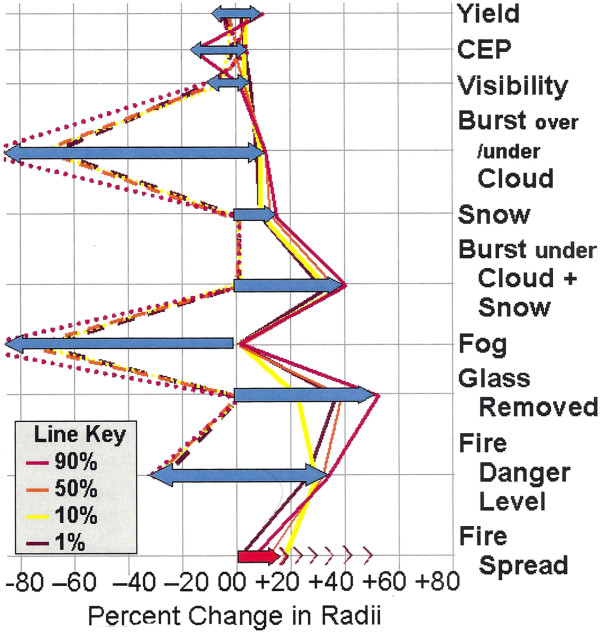
Sensitivity of Fire Radii to different Values of Model Variables.

Binninger states “The difference between risk and uncertainty is that risks have, or could have, known probability distributions, uncertainties cannot. The risk is quantified by the variability used to define the probability distribution” [28] Binninger goes on to state “there is no foolproof way short of experimentation to provide guarantees about the scale of errors introduced by factors that we don’t know.” We can discuss the likely influence of simplifications introduced from factors we know conceptually but don’t know operationally or experimentally. We know the effects of nuclear detonations on buildings in the desert or on Nagasaki or Hiroshima but we do not know the result of a nuclear explosion in a major U.S. downtown with many very high concrete and steel buildings. A summary and comparison of uncertainties in the radii of fires from a nuclear detonation with 1, 10, 50 and 90% radii error probabilities is presented in Figure 1 of this paper, taken from Binninger’s Figure twenty-four. These and further uncertainties are discussed in detail in Binninger [28]. In our calculations we have assumed that we know the yield, the circular error of probability (CEP) is 0 (where we detonate the bomb), visibility is typical for that location in that season, the detonation is below any cloud, there is no snow, fog, prior glass breakage and the fire danger level is average. Fire spread was unknown. For a further discussion of fire spread as opposed to ignition from fires see Binninger [28]. Therefore, the range of variables and the uncertainties associated with them are amply discussed in these sources, and relatively in Figure 1 of this manuscript. Given the additional uncertainties of the building construction in these nations, the additional error from these model uncertainties are not likely to substantially affect the medical outcomes in these simulations.

In short, our model results use typical values for variables and should yield good results most of the time. There will be occasions when the results for thermal fluence will differ substantially from the average such as snow on the ground with thick cloud above the fireball (gross underestimation) or with the fireball above low cloud and fog (overestimation) but we assume a detonation would not take place during such adverse conditions. Results for radiation and blast are typical of the best available non- classified models but clearly have not been experimentally verified for large urban downtown areas with many tall concrete buildings. Supportive of this approach is the factor that the large glass surfaces of modern urban buildings would increase reflectance, prior to collapsing from the blast.

## Results and discussion

### Effects of single nuclear weapon detonations in Iran

**Figure 2:**
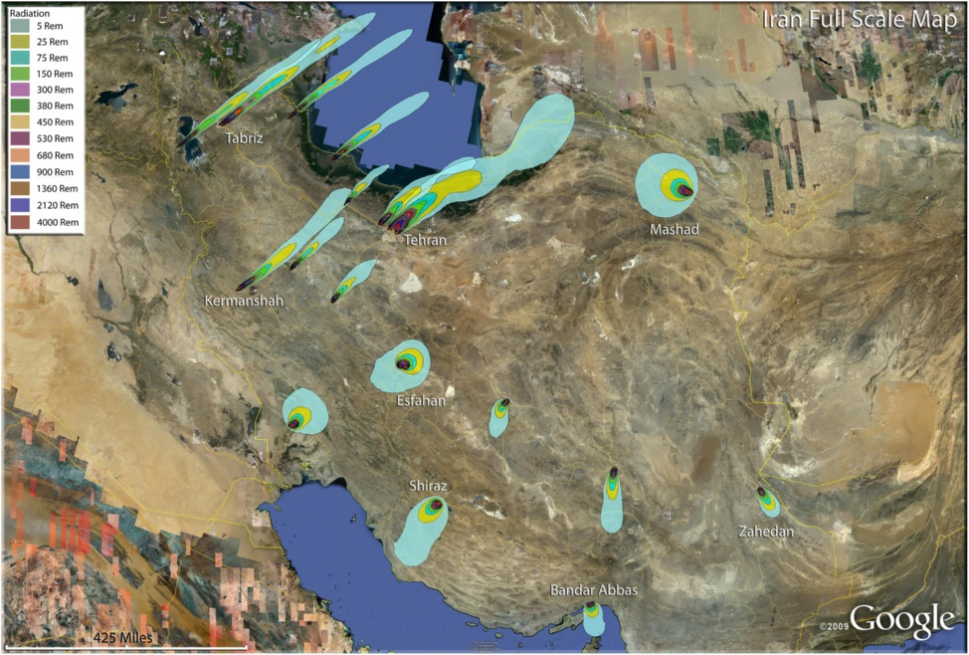
**Iran – Map of all modeled detonations.** Showing the radiation exposure plumes from all of the nuclear detonations nationwide for this scenario.

**Figure 3:**
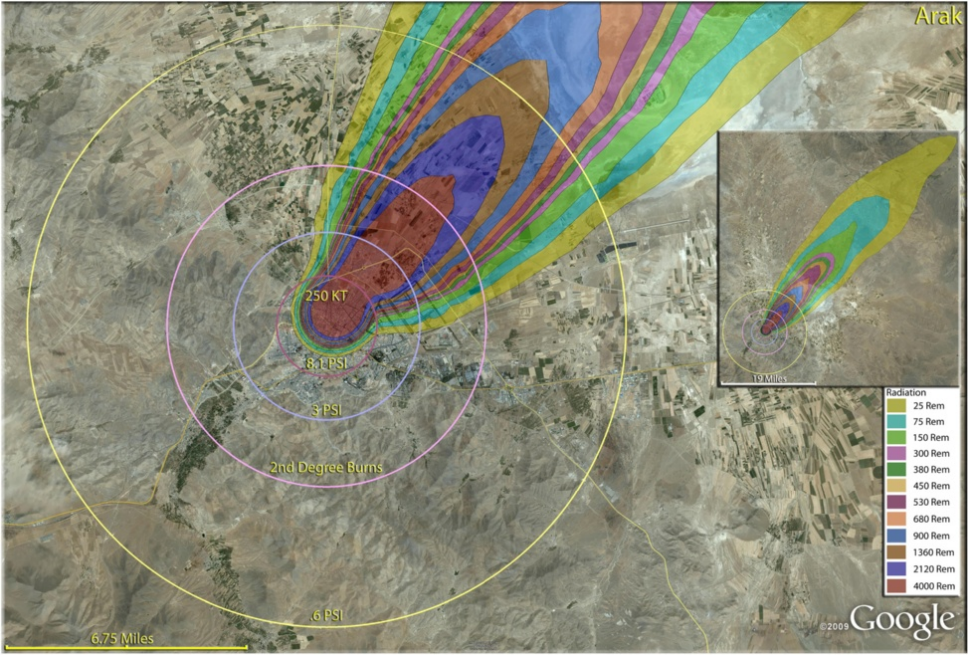
**Single 250 KT nuclear weapon detonation casualties for Arak, Iran.** Rings display trauma causalities for 8.1 and 3.0 psi, 2nd degree burns, and the outer ring is lower trauma casualties for 0.6 psi. Radiation exposures are delineated by color in the dispersion plume.

**Figure 4:**
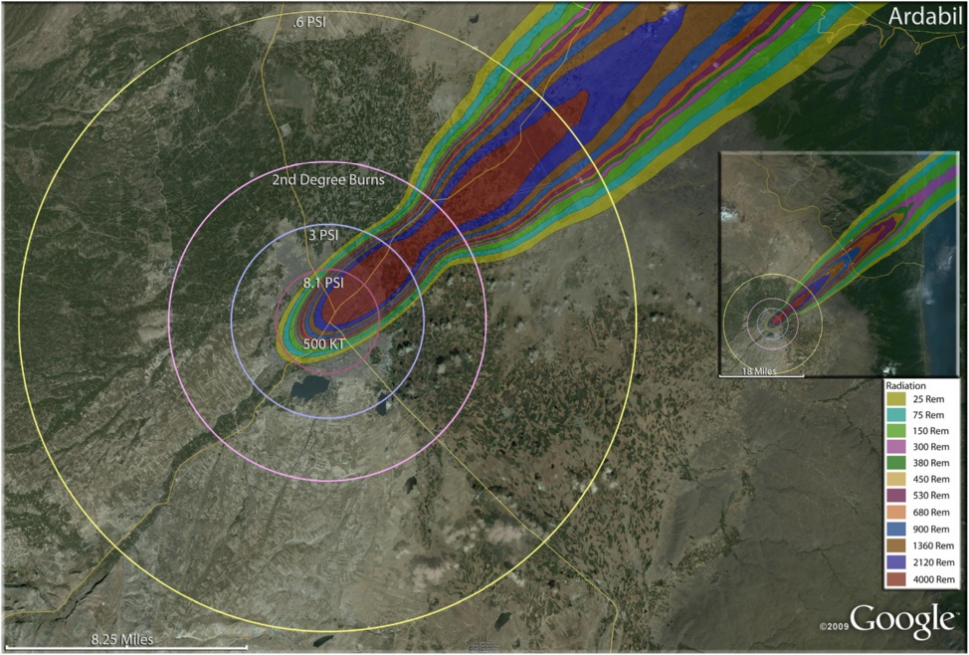
**Single 500 KT nuclear weapon detonation casualties for Ardabil, Iran.** Rings display trauma causalities for 8.1 and 3.0 psi, 2nd degree burns, and the outer ring is relatively lower trauma casualties for 0.6 psi. Radiation exposures are delineated by color in the dispersion plume.

**Figure 5:**
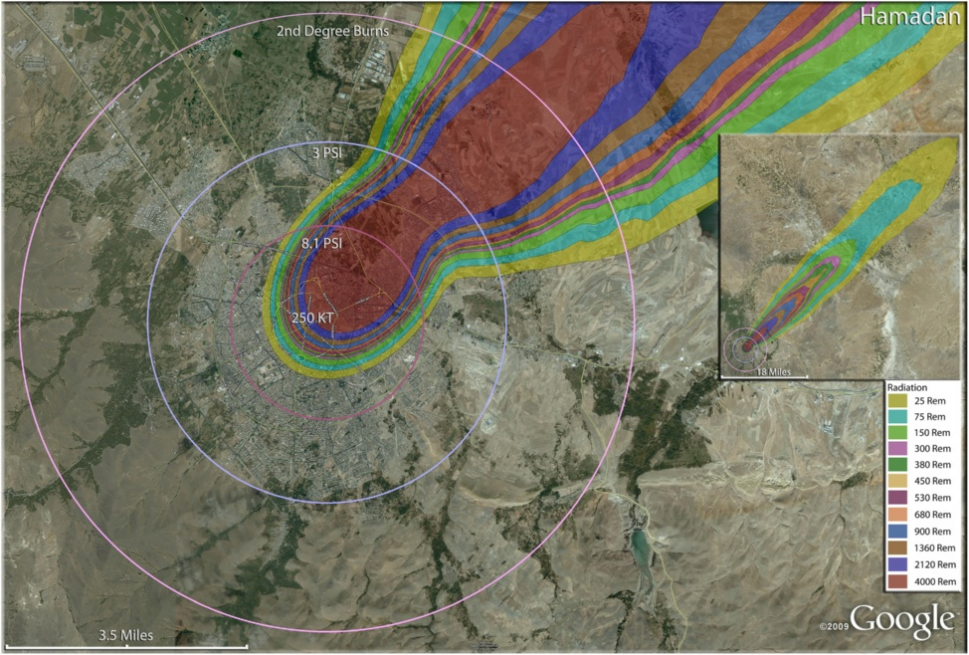
**Single 250 KT nuclear weapon detonation casualties for Hamandan, Iran.** Rings display trauma causalities for 8.1 and 3.0 psi, and 2nd degree burns. Radiation exposures are delineated by color in the dispersion plume.

**Figure 6:**
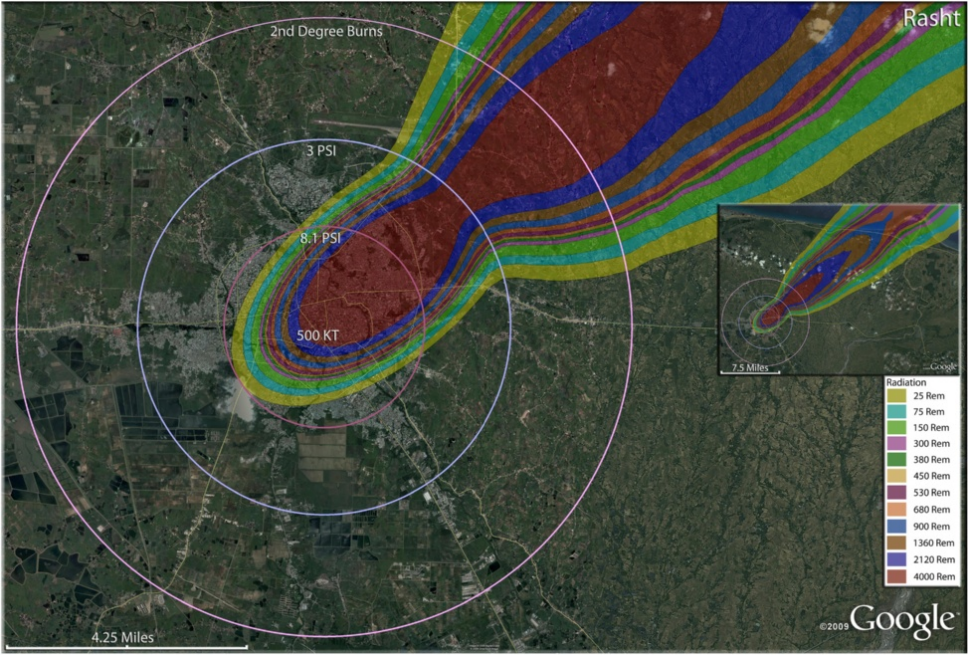
**Single 500 KT nuclear weapon detonation casualties for Rasht, Iran.** Rings display trauma causalities for 8.1 and 3.0 psi, and 2nd degree burns. Radiation exposures are delineated by color in the dispersion plume.

**Figure 7:**
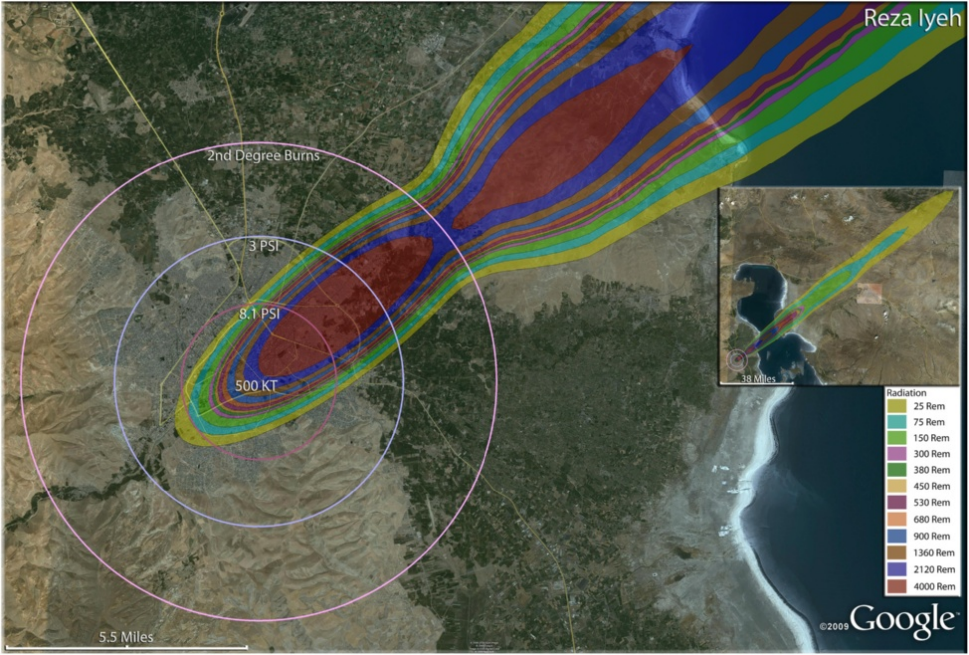
**Single 500 KT nuclear weapon detonation casualties for Reza Iyeh, Iran.** Rings display trauma causalities for 8.1 and 3.0 psi, and 2nd degree burns. Radiation exposures are delineated by color in the dispersion plume.

**Figure 8:**
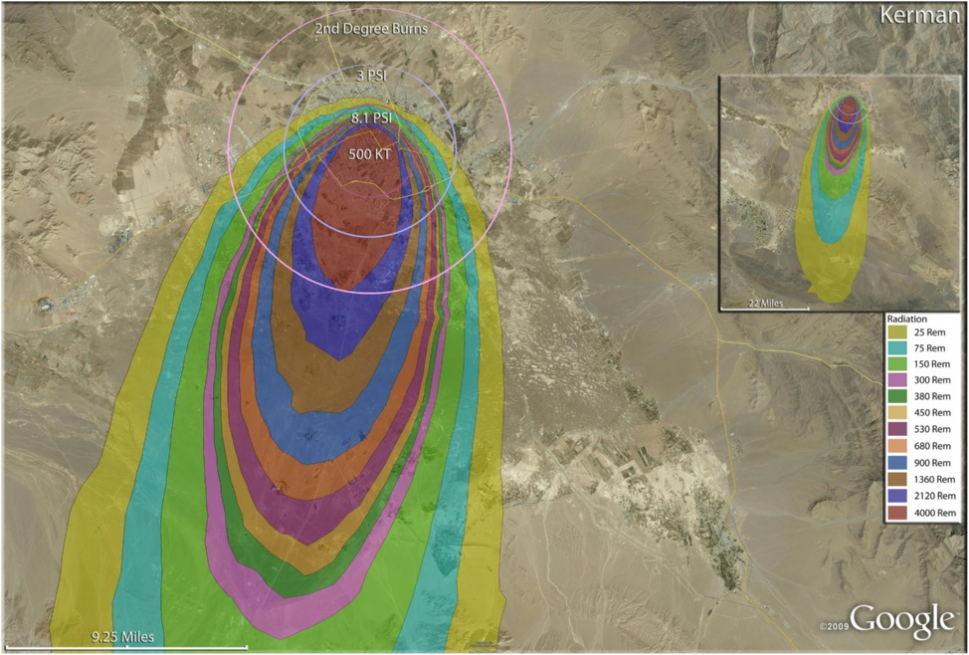
**Single 500 KT nuclear weapon detonation casualties for Kerman, Iran.** Rings display trauma causalities for 8.1 and 3.0 psi, and 2nd degree burns. Radiation exposures are delineated by color in the dispersion plume.

**Figure 9:**
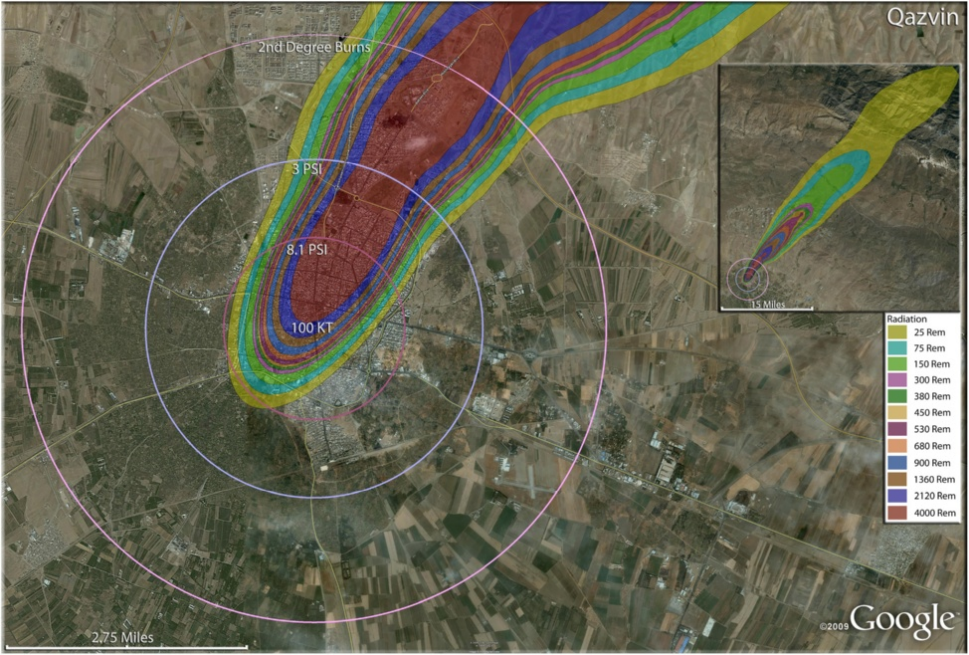
**Single 100 KT nuclear weapon detonation casualties for Qazvin, Iran.** Rings display trauma causalities for 8.1 and 3.0 psi, and 2nd degree burns. Radiation exposures are delineated by color in the dispersion plume.

**Figure 10:**
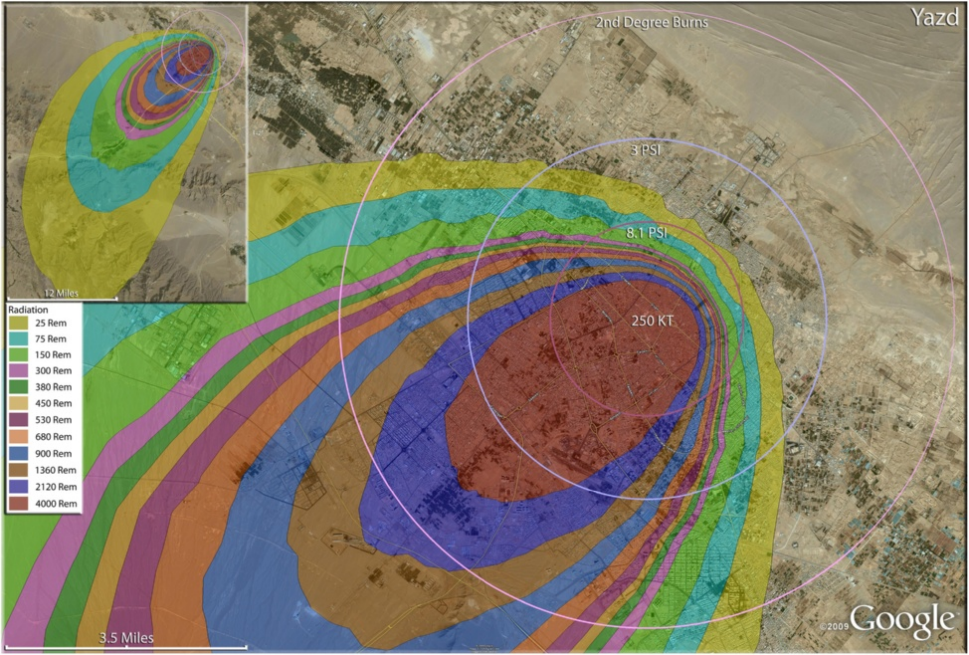
**Single 250 KT nuclear weapon detonation casualties for Yazd, Iran.** Rings display trauma causalities for 8.1 and 3.0 psi, and 2nd degree burns. Radiation exposures are delineated by color in the dispersion plume.

**Figure 11:**
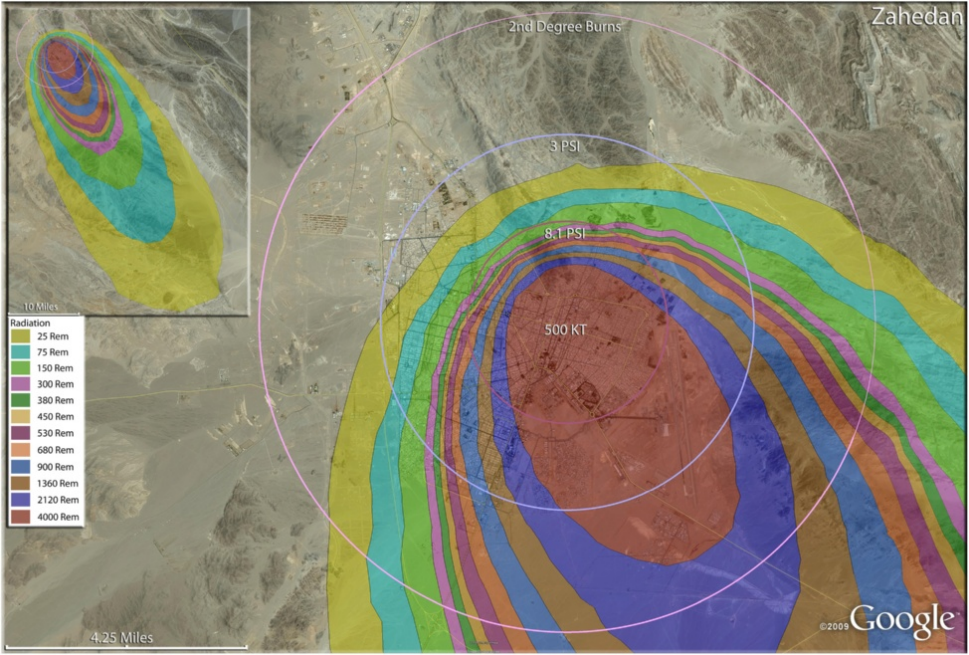
**Single 500 Kt nuclear weapon detonation casualties for Zahedan, Iran.** Rings display trauma causalities for 8.1 and 3.0 psi, and 2nd degree burns. Radiation exposures are delineated by color in the dispersion plume.

Medical casualty simulations are presented as the consequence of nuclear detonations for 18 cities in Iran (Figure 2). At the nation-wide scale of this illustration, only the radiation casualty distributions are seen, with the trauma and thermal casualties not visible at this level of resolution. It should be noted that these cigar-shaped plumes are based on flat, open terrain and steady winds and stable meteorological conditions. Wind direction often varies with altitude. It should be expected that the actual fallout distribution could vary considerably from these diagrams, with various irregular shapes due to the micrometeorology and terrain variables. It is evident that significant radiation casualties result over a hundred miles away from the detonation points. Different wind directions for the September winds are also evident in the distant reaches of this large country. Depending on the size of the Iranian cities, nine of the major urban areas would endure maximal devastation with a single medium-sized nuclear device. In a demonstration of weapon size relative to urban population distribution, either a 100, 250, or 500 Kt nuclear weapon was utilized in each simulation, with the purpose of showing coverage of inhabited areas by blast, thermal, and radiation casualties. These single weapon cities are presented in Figures 3, 4, 5, 6, 7, 8, 9, 10 and 11.

As weather and atmospheric conditions have significant effects on the outcomes of nuclear detonations [14], the distinctive climatic conditions in the Middle East can be expected to alter the quantity and distribution of the resulting mass casualties. Bursts occurring near the surface will create large amounts of dusts from the ground and destroyed buildings. Dust loads in the air of Iran are going to be significantly higher than in the U.S., so thermal and radiation induced casualties could be affected by the inherently higher airborne dust concentrations to which nuclear-generated dusts are being added. The higher dust loads also would impact on the “dirtiness” of wound care in Iranian detonation aftermaths, which would be dramatic in any post-nuclear war medical treatment environment [30]. It was assumed that the generally accepted figure of 15% of the population was outdoors at the time of detonation.

The simulation for Arak (Figure 3), using a 250 Kt weapon, provides the basic template for medical mass casualties resulting from nuclear detonations. The blast damage from three levels of overpressure, 8.1 psi, 3 psi and 0.6 psi are provided in concentric rings. The areas where people would experience second degree burns are indicated inside the pink ring. The regions burdened with radiation victims are indicated by colored plumes, with fatal radiation doses received by people well beyond the second degree burn regions, and even beyond the areas affected by broken glass within the 0.6 psi ring. In the inset, it can be seen that radiation levels sufficient to cause death and serious injury extend far out into the desert. In Arak, there would be 387,000 fatalities, or 93% of the total population (Table 4). The injury pattern in Arak was repeated for most Iranian cities, where the thermal injuries outnumbered the blast casualties, and radiation injuries were only a small fraction of all casualties (Tables 5 and 6).

**Table 4:** Total casualties for all scenarios

**Scenario/City**	**Weapon yield (kt)**	Estimated population*	Total* fatalities	Total* injuries
**Israeli Cities**				
- Beer Sheva	15	208,770	105,510	35,090
- Haifa	15	323,890	69,420	50,400
- Tel Aviv (double strike)	Dual 15	1,372,440	229,330	147,340
**Iranian Single-Strike cities**				
- Arak	250	424,270	387,600	32,240
- Ardabil	500	456,500	428,120	22,240
- Hamadan	250	386,130	362,400	35,250
- Karaj	15	1,125,360	157,960	130,960
- Karaj	50	1,125,360	325,860	199,270
- Karaj	100	1,125,360	508,030	219,070
- Karaj	250	1,125,360	744,100	210,460
- Karaj	500	1,125,360	891,190	164,770
- Kerman	500	560,320	510,850	36,110
- Qazvin	100	460,250	423,200	38,190
- Rasht	500	503,140	482,940	46,450
- Reza Iyeh	500	582,820	545,450	44,170
- Yazd	250	435,120	326,610	48,930
- Zahedan	500	602,530	578,950	23,330
**Iranian Multiple-Strike cities**	**Yield/Number of weapons**			
- Ahvaz	500 + 250	1,050,530	852,140	74,330
- Bandar Abbas	100 × 3	467,510	438,240	22,160
- Esfahan	500 × 2	1,836,920	1,510,050	199,640
- Kermanshah	250 × 3	751,710	718,480	33,020
- Mashad	500 × 3	2,242,760	2,178,020	59,250
- Shiraz	500 × 2	1,227,820	1,037,170	133,190
- Tabriz	500 × 2	1,264,550	1,220,250	73,760
- Tehran	100 × 5	8,317,080	3,615,350	1,622,360
- Tehran	250 × 5	8,317,080	5,594,200	1,577,220
- Tehran	500 × 5	8,317,080	7,127,800	791,080

*Total casualties plus uninjured do not equal estimated population, as direct blast effects and fallout plumes typically extend beyond the areas of highest population density and may include other communities.

**Table 5:** Injuries due to radiation by cities

		**Prompt radiation (rad)**		**Fallout exposure (rem)**	
**Scenario/City**	**Weapon yield (Kt)**	**600**	**300**	**680**	**300**
**Israeli Cities**					
- Beer Sheva	15	930	2,810	5,970	1,800
- Haifa	15	460	1,410	9,370	1,930
- Tel Aviv (double strike)	Dual 15	1,490	4,520	78,900	25,360
**Iranian Single-Strike cities**					
- Arak	250	680	570	3,100	1,810
- Ardabil	500	920	210	3,220	1,250
- Hamadan	250	650	620	2,410	650
- Karaj	15	1,050	4,020	11,310	2,450
- Karaj	50	1,240	1,330	6,180	2,210
- Karaj	100	800	1,370	5,610	3,380
- Karaj	250	630	800	4,780	1,690
- Karaj	500	600	350	7,620	3,250
- Kerman	500	250	190	3,360	3,820
- Qazvin	100	1,310	1,280	10,340	4,840
- Rasht	500	750	230	10,000	1,190
- Reza Iyeh	500	790	250	4,480	1,370
- Yazd	250	260	350	12,210	6,960
- Zahedan	500	190	180	4,720	3,230
**Iranian Multiple-Strike cities**	**Yield/Number of weapons**				
- Ahvaz	500 + 250	80	130	35,060	15,190
- BandarAbbas	100 × 3	620	1,060	9,500	5,820
- Esfahan	500 × 2	30	20	104,500	46,580
- Kermanshah	250 × 3	13,480	4,090	3,030	1,530
- Mashad	500 × 3	50	20	48,990	4,990
- Shiraz	500 × 2	40	60	26,230	27,620
- Tabriz	500 × 2	2,530	610	6,320	2,160
- Tehran	100 × 5	5,440	7,790	602,030	143,020
- Tehran	250 × 5	3,990	3,890	840,510	36,770
- Tehran	500 × 5	2,580	1,340	211,100	23,320

**Table 6:** Injuries due to burns and blast by cities

		**Burn and mass fire zones**			**Blast overpressure (psi)**			
**Scenario/City**	**Yield (Kt)**	**50% mass fire**	**3rd- degree burns**	**2nd- degree burns**	**8.1**	**4.9**	**3**	**0.6**
**Israeli Cities**								
- Beer Sheva	15	13,530	21,010	11,680	130	710	11,600	22,600
- Haifa	15	7,650	13,710	17,430	80	330	6,260	36,200
- Tel Aviv	Dual 15	21,840	39,680	39,060	250	1,100	18,460	115,770
**Iranian Single-Strike cities**								
- Arak	250	13,810	27,580	1,340	2,000	6,100	11,540	12,160
- Ardabil	500	6,000	16,070	1,010	2,550	4,400	5,350	7,350
- Hamadan	250	13,610	29,680	1,860	2,080	7,720	9,270	15,140
- Karaj	15	22,450	37,290	49,600	150	780	18,700	111,150
- Karaj	50	62,680	106,220	57,430	640	3,890	39,180	155,450
- Karaj	100	106,910	168,240	24,900	830	7,240	55,850	154,890
- Karaj	250	110,720	167,450	15,710	2,570	15,700	71,720	118,610
- Karaj	500	55,410	121,240	10,220	4,660	23,020	50,570	85,820
- Kerman	500	11,130	32,590	1,270	2,380	11,320	14,130	8,160
- Qazvin	100	17,630	32,350	1,790	1,340	4,540	10,130	21,980
- Rasht	500	10,560	26,710	1,970	2,970	6,700	9,260	21,520
- Reza Iyeh	500	12,440	34,490	2,370	3,210	10,760	13,280	16,130
- Yazd	250	20,500	35,570	5,070	1,080	4,610	12,500	30,360
- Zahedan	500	6,760	22,920	100	2,090	8,610	11,510	1,120
**Iranian Multiple- Strike Cities**	**Yield/Number of weapons**							
- Ahvaz	250 + 500	38,180	55,270	6,530	210	3,800	6,850	63,270
- Bandar Abbas	100 × 3	11,970	21,060	400	650	3,880	7,810	9,750
- Esfahan	500 × 2	86,520	140,960	23,690	320	6,970	51,710	139,080
- Kermanshah	250 × 3	9,420	64,540	12,450	20,070	26,430	41,900	102,230
- Mashad	500 × 3	26,950	51,360	3,260	470	3,890	16,380	38,110
- Shiraz	500 × 2	57,360	107,560	16,140	1,070	9,750	36,220	86,090
- Tabriz	500 × 2	17,070	62,310	1,370	8,020	25,510	19,130	16,000
- Tehran	100 × 5	469,720	846,470	231,010	5,650	37,770	249,910	1,325,520
- Tehran	250 × 5	419,030	803,370	299,430	13,090	60,030	280,380	1,221,110
- Tehran	500 × 5	309,110	591,320	50,680	16,000	73,560	192,590	485,110

In the larger city of Ardabil (Figure 4), a 500 Kt weapon shows that the widely distributed population can be almost completely reached by this larger-yield device (Figure 4). Indeed, blast and thermal casualties are likely to encompass almost the entire inhabited area around the city. As with Arak, fatalities would result in the majority of the population. Indeed, fatalities and injuries together (total casualties) would include 98.6% of the population of Ardabil. The 250 Kt detonation for Hamadan (Figure 5) reveals results similar to Ardabil and Arak. The great majority of the radiation plume in fact lies outside these inhabited areas, as does much of the thermal energy and overpressure sufficient to cause glass injuries. It is noteworthy that the fallout plumes from the Ardabil and Arak detonations reach the coastal areas of the Caspian Sea with sufficient radioactivity to induce serious radiation casualties. Fortunately for the coastal areas, which tend to be more heavily populated than the widespread inland arid regions of the country, the relatively narrow width of the plume results in only a short stretch of the coastal area actually being affected.

This “coastal effect” is also seen with the simulations for Rasht (Figure 6) and Reza Iyeh (Figure 7), where severe radiation casualties will be seen in the coastal areas, but in a limited arc relative to the entire coastal area. In this way, the narrow population distribution along the coastal areas results in a somewhat diminished exposure to these radiation plumes from inland urban areas. However, radiation casualties from the Reza Iyeh detonation would extend even across the Caspian Sea to the Turkmenistan coastal areas on the other side of the inland sea (Figure 7 inset).

The largest of the weapons examined in this study, 500 Kt, resulted in similarly high fatality rates in Kerman (Figure 8). As in other Iranian cities, the combination of fatality and injury outcomes of the compact, crowded urban areas resulted in a very small proportion of the residents, 3.4% of the population in the case of Kerman, surviving without injury. Over 32,000 of the city’s residents will have third degree burns (Table 4). Kerman had one of the higher number of trauma casualties resulting from greater than 3psi, at 27,830. In contrast, Kerman had one of the smaller number of radiation fallout victims receiving >300 rem, at about 7,000. The slightly smaller city of Qazvin (Figure 9) had a similar number of third degree burn victims as Kerman, even though the weapon used was only one fifth the size (100 Kt). By contrast, the trauma casualties from pressures >3psi were only 16,000 in Qazvin (Table 4).

One of the lower fatality rates seen in the single nuclear weapon strikes was at Yazd (Figure 10), where 75% of the city’s population were fatalities following a 250 Kt detonation. The proportion of injuries there was almost twice as high in Yazd relative to other first strike cities, at about 11% of the total population. The impact of geography on the medical distribution of casualties can be discerned to some extent in Zahedan (Figure 11), where the compact nature of Iranian urban population distribution is seen. This compressed nature of Iranian urban sprawl (essentially the lack of urban sprawl as seen in American and European cities) resulted in a relatively much higher percentage of fatalities due to trauma and thermal burn injuries. Therefore, this also results concurrently in a relatively small percentage of the population (about 2%) receiving >300 rem in radiation from prompt and fallout sources. Essentially the compressed cities like Zahedan have populations dying of trauma and burns rather than from radiation. No doubt the compressed population characteristics also contributed to the stunning 96% fatality rate in Zahedan.

### Thermal burn casualties

It has been noted in previous publications by this group that the mass thermal burn casualties expected with nuclear weapons are going to be very difficult to treat [1,2]. Typical burn care management involves a high ratio of medical personnel to patients, which can be accommodated when there are only a few burn victims at a time. With tens of thousands (or more) of burn victims simultaneously, however, burn care becomes difficult, and virtually nonexistent, in nuclear war scenarios. There is a dramatic difference in the number of second degree burn casualties relative to third degree burn and mass fire burn injuries, with second degree burn casualties considerably smaller than the other burn categories. In Hamadan, as a typical example, there were approximately 2,000 second degree thermal burn victims to 30,000 and 14,000, respectively, for third degree and mass fire burn casualties.

Third degree burn victims generally ranged from 20,000 to 35,000 for single strike cities in Iran, but larger cities with single and multiple strikes produced hundreds of thousands of third degree burn victims. This preponderance of third degree burn victims is a very difficult outcome for the emergency response communities of any nation. It is likely that these thousands of thermal burn victims will receive little to no care, as the very limited surviving medical resources are most likely to be devoted to the trauma casualties, which are more familiar to medical personnel and require relatively less effort per patient [31,32].

### Relative casualty impacts of different nuclear weapon yields

With the advent of nuclear war in dense, compact cities in the Middle East, the actual impact of nuclear yield is an interesting and perhaps surprising issue. As nuclear powers expand their nuclear capability over time, they gradually develop higher yields in their weapons. For the United States and the Soviet Union, this development occurred rapidly, going from approximately 15 Kt weapons in the 1940s to 500 Kt (and larger) devices in the 1950s. For nations with fewer resources available for nuclear weapon development, this process can take much longer. After being a nuclear power for over 20 years, Pakistan is still fielding 15 Kt weapons, though the number of weapons continues to increase steadily (now believed to between 80 and 90 weapons). It is speculated that Israel has been able to develop at least up to 500 Kt weapons over the last 40 years. The larger weapons play a dramatic role in casualty propagation in Middle East cities, though it appears that the increase in effect is not linear, especially for thermal casualties.

**Figure 12:**
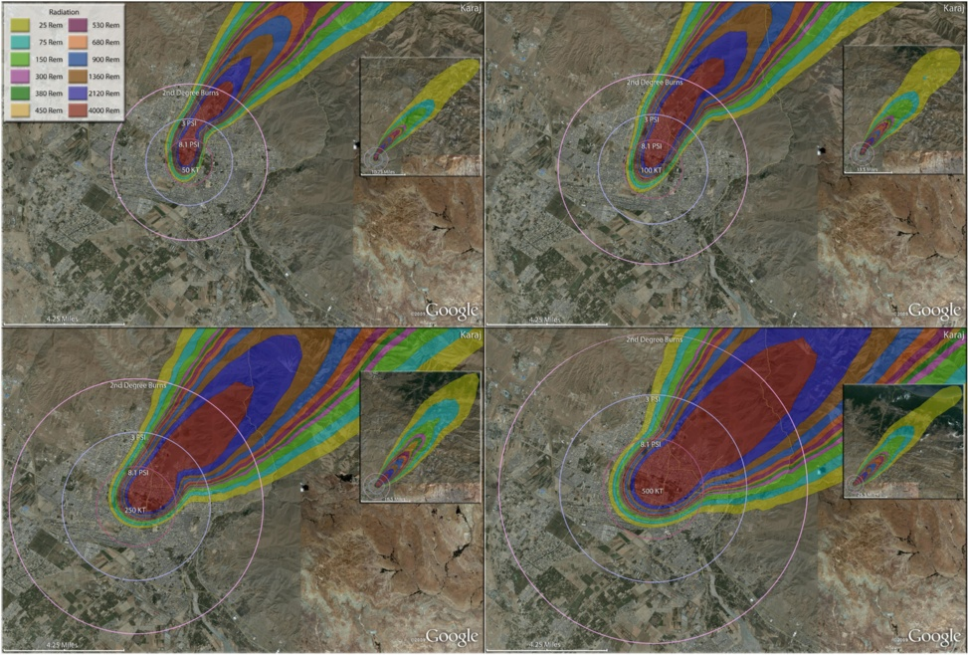
**Multiple detonation casualties for one 50 Kt, one 100 Kt, one 250 Kt, one 500 Kt nuclear weapon, Karaj, Iran.** Rings display trauma causalities for 8.1 and 3.0 psi, and 2nd degree burns. Radiation exposures are delineated by color in the dispersion plume.

In Figure 12, the relative impact of different weapon sizes (50, 100, 250, and 500 Kt) on casualty outcomes can be seen on the city of Karaj. It can be seen that fatalities increase with weapon size, though not linearly. When the yield was increased by a factor of 3.3 from 15 Kt to 50 Kt it results in an approximate two-fold increase in fatalities (Table 4). The diminishing impact of further increase in yield is illustrated by the 46% fatality increase with a 250% increase in yield from 100 Kt to 250 Kt, and 20% fatality increase with the 100% yield increase from 250 Kt to 500 Kt. Due to the overwhelming proportion of casualties being fatalities, after 100 Kt the approximate number of injuries is about the same, and actually decrease somewhat for a 500 Kt detonation. There is an approximately linear increase in mass fire and third degree burn casualties from the smallest weapon, 15 Kt, to the 50 Kt weapon, though not for second degree burn injuries (Tables 5 and 6).

### Casualty distributions for multiple nuclear weapons in one city

**Figure 13:**
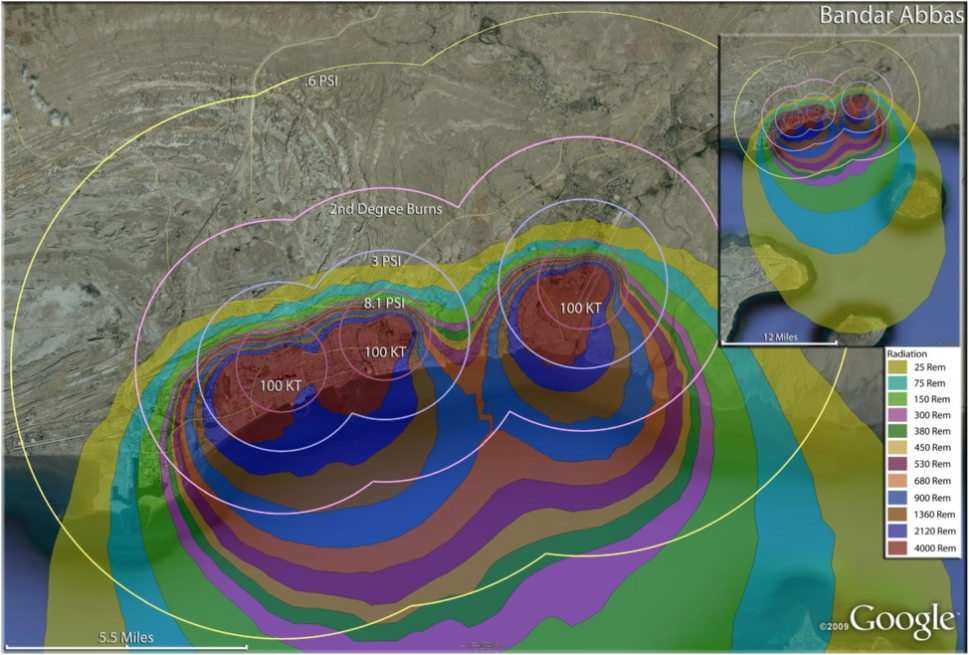
**Multiple detonation casualties for three 100 Kt nuclear weapons for Bandar Abbas, Iran.** Rings display trauma causalities for 8.1 and 3.0 psi, 2nd degree burns, and the outer ring is lower trauma casualties for 0.6 psi. Radiation exposures are delineated by color in the dispersion plume.

**Figure 14:**
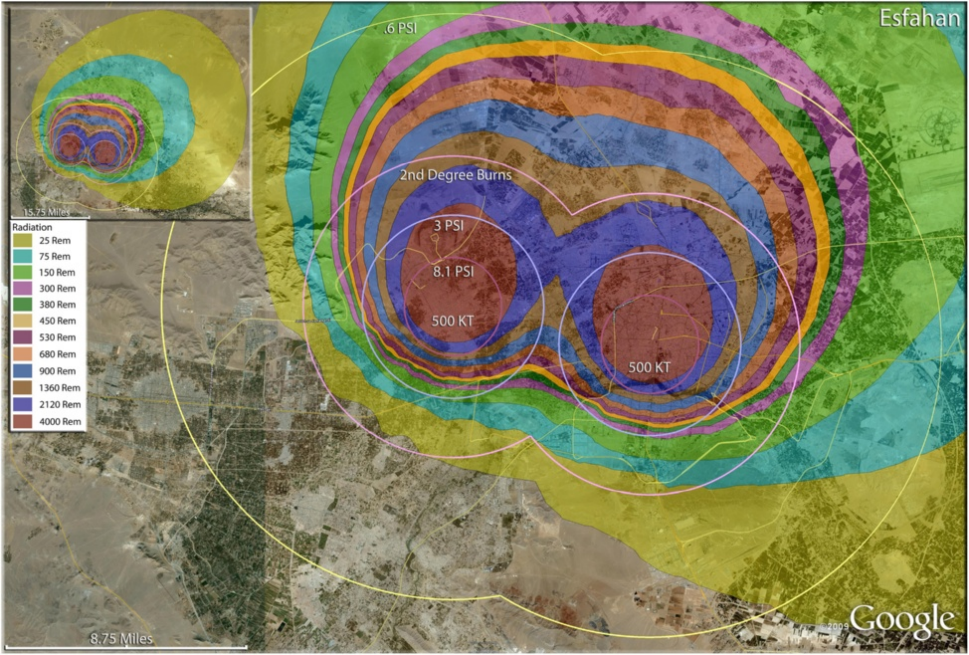
**Multiple detonation casualties for two 500 Kt nuclear weapons for Esfahan, Iran.** Rings display trauma causalities for 8.1 and 3.0 psi, 2nd degree burns, and the outer ring is lower trauma casualties for 0.6 psi. Radiation exposures are delineated by color in the dispersion plume.

**Figure 15:**
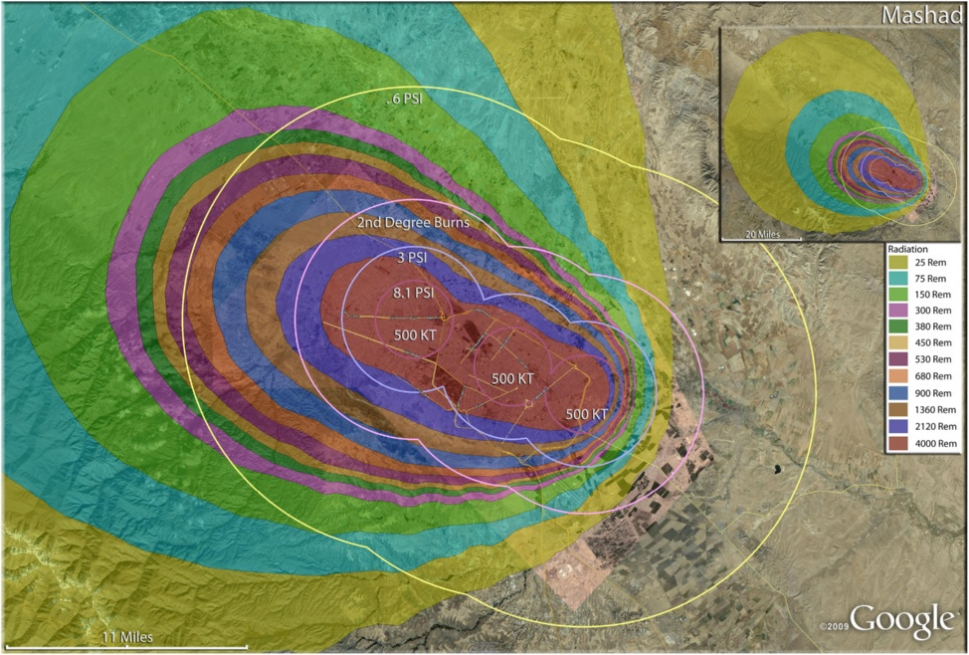
**Multiple detonation casualties for three 500 Kt nuclear weapons for Mashad, Iran.** Rings display trauma causalities for 8.1 and 3.0 psi, 2nd degree burns, and the outer ring is lower trauma casualties for 0.6 psi. Radiation exposures are delineated by color in the dispersion plume.

**Figure 16:**
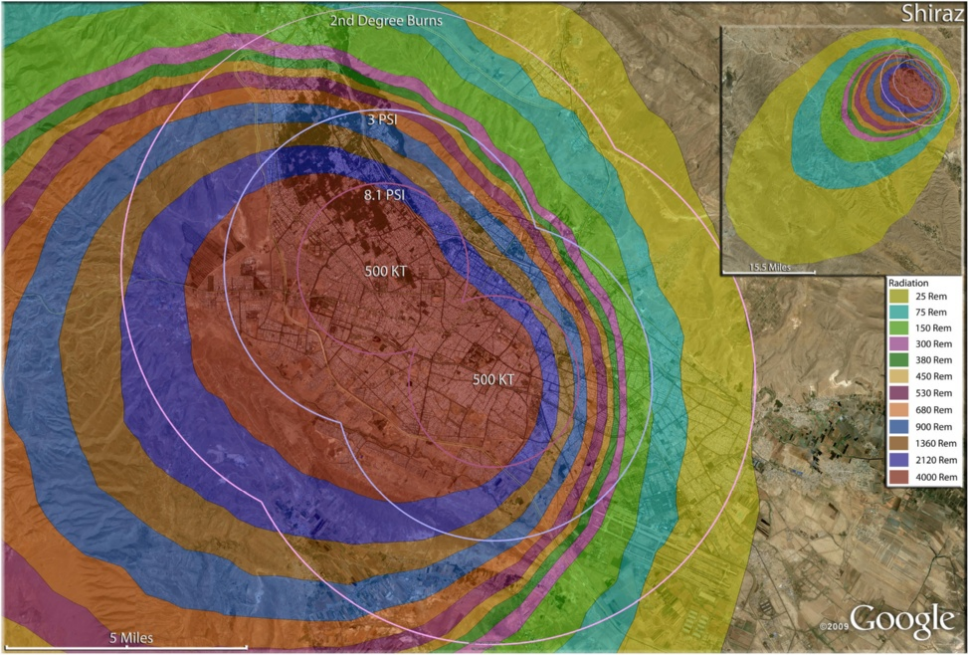
**Multiple detonation casualties for two 500 Kt nuclear weapons for Shiraz, Iran.** Rings display trauma causalities for 8.1 and 3.0 psi, and 2nd degree burns. Radiation exposures are delineated by color in the dispersion plume.

**Figure 17:**
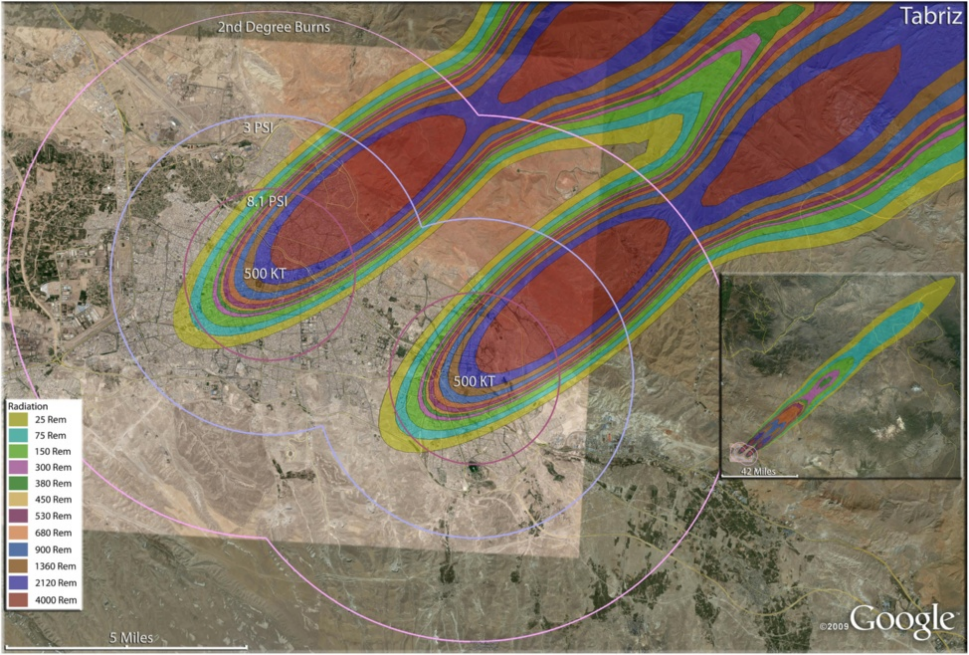
**Multiple detonation casualties for two 500 Kt nuclear weapons for Tabriz, Iran.** Rings display trauma causalities for 8.1 and 3.0 psi, and 2nd degree burns. Radiation exposures are delineated by color in the dispersion plume.

**Figure 18:**
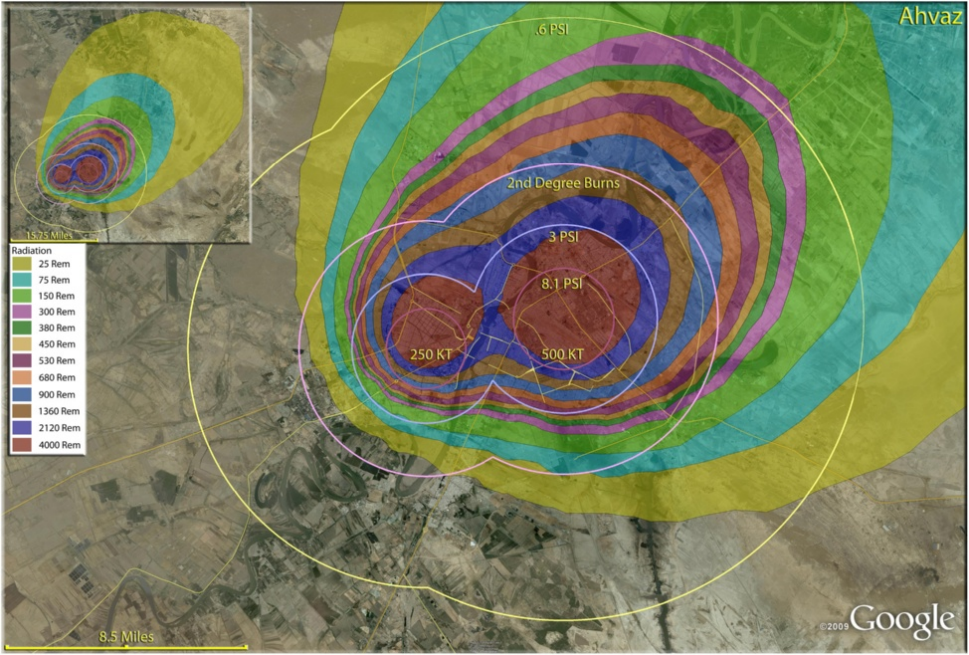
**Multiple detonation casualties for one 250 Kt and one 500 Kt nuclear weapon for Ahvaz, Iran.** Rings display trauma causalities for 8.1 and 3.0 psi, 2nd degree burns, and the outer ring is lower trauma casualties for 0.6 psi. Radiation exposures are delineated by color in the dispersion plume.

**Figure 19:**
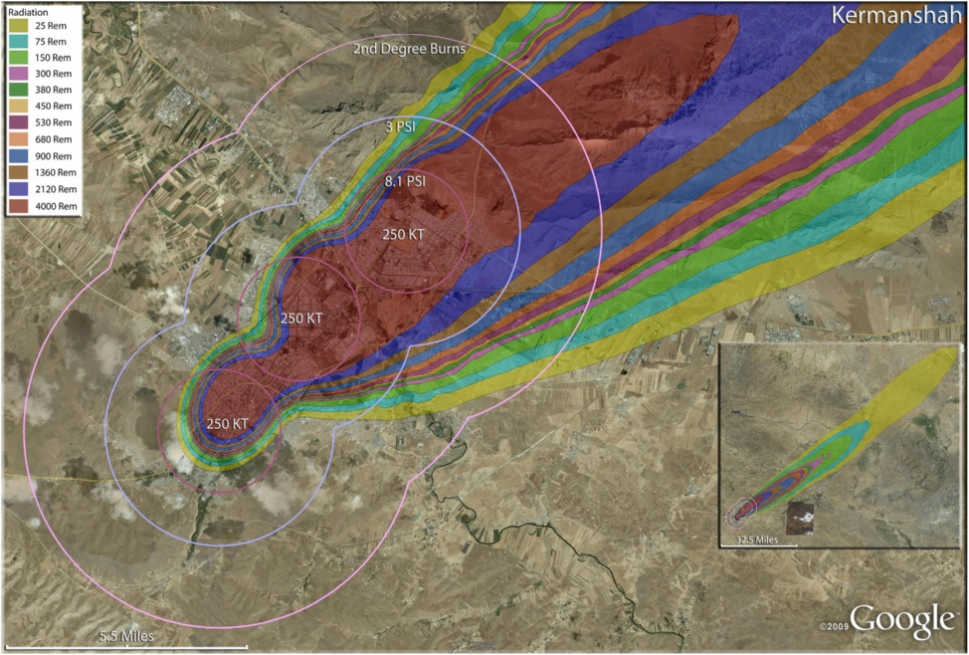
**Multiple detonation casualties for three 250 Kt nuclear weapons Kermanshah, Iran.** Rings display trauma causalities for 8.1 and 3.0 psi, and 2nd degree burns. Radiation exposures are delineated by color in the dispersion plume.

**Figure 20:**
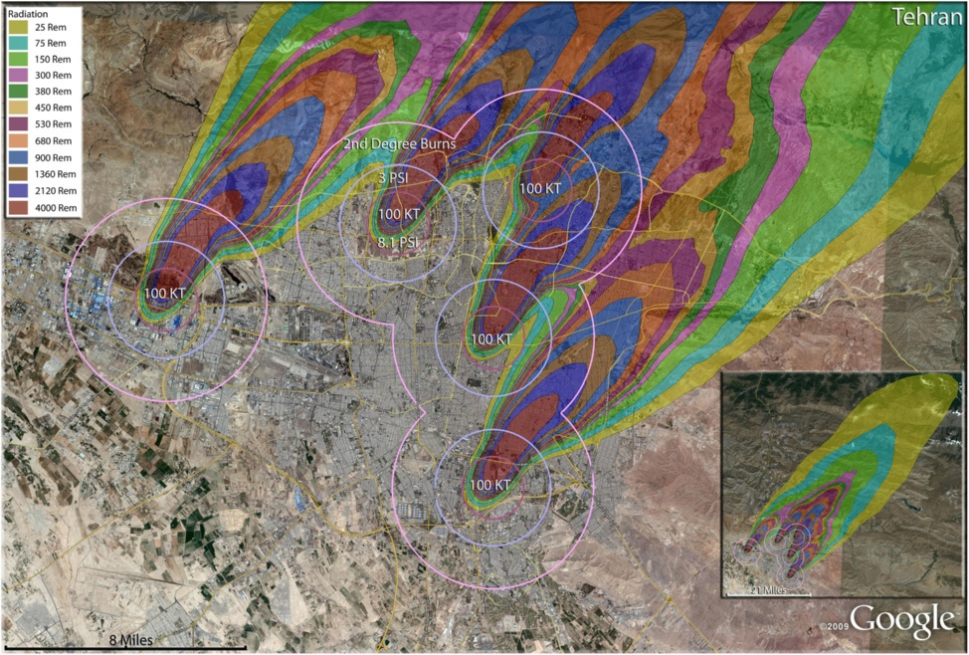
**Multiple detonation casualties for five 100 Kt nuclear weapons for Tehran, Iran.** Rings display trauma causalities for 8.1 and 3.0 psi, and 2nd degree burns. Radiation exposures are delineated by color in the dispersion plume.

**Figure 21:**
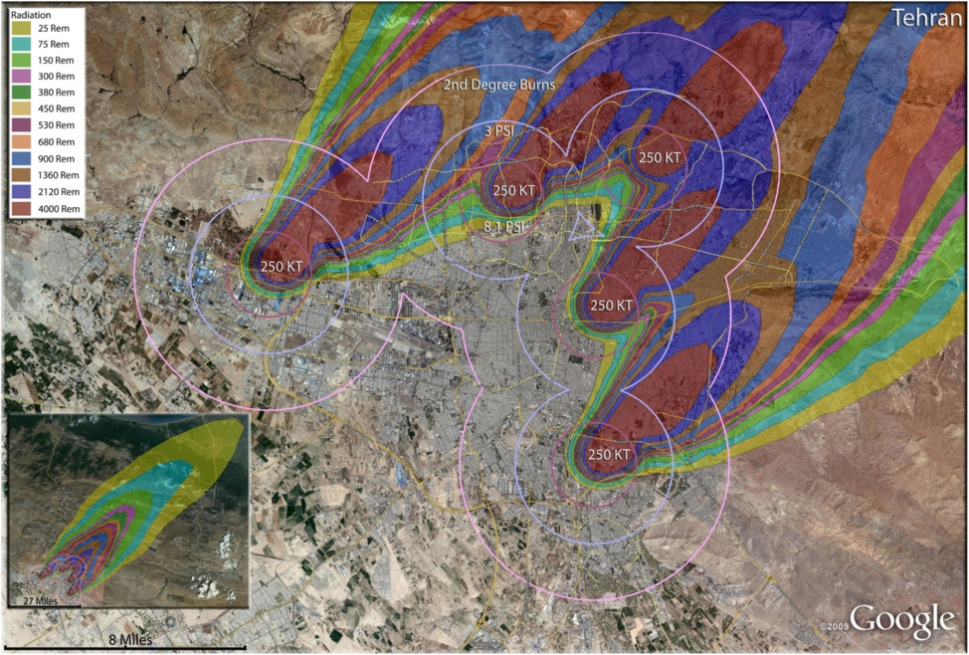
**Multiple detonation casualties for five 250 Kt nuclear weapons for Tehran, Iran.** Rings display trauma causalities for 8.1 and 3.0 psi, and 2nd degree burns. Radiation exposures are delineated by color in the dispersion plume.

**Figure 22:**
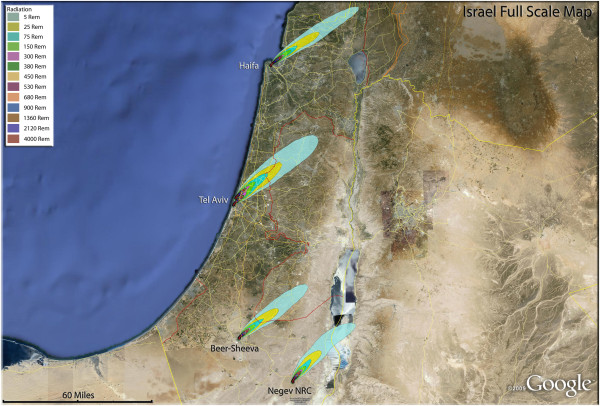
**Multiple detonation casualties for five 500 Kt nuclear weapon for Tehran, Iran.** Rings display trauma causalities for 8.1 and 3.0 psi, and 2nd degree burns. Radiation exposures are delineated by color in the dispersion plume.

Once a nation reaches the technological threshold of the mass production of relatively high yield nuclear weapons, the potential outcomes for urban areas of their enemies is bleak. In this analysis, the speculation of the deployment of a number of large nuclear weapons on Iranian cities resulted in a terrifyingly efficient coverage of the populations with casualties, particularly with fatalities. The results of multiple strikes with nuclear weapons on a single city are shown in Figures 12, 13, 14, 15, 16, 17, 18, 19, 20, 21 and 22. Distinctive evidence of the destructive capacity of relatively smaller nuclear weapons is demonstrated by the three 100 Kt weapon strikes on Bandar Abbas, the seaport on the Persian Gulf at the geopolitically strategic Strait of Hormuz (Figure 13). A staggering 94% of the population would be fatalities in this scenario, with a mere 1% escaping some form of injury. An interesting aspect of this coastal community is the appearance of over 21,000 third degree burn casualties, and only a few hundred second degree burn victims, probably due to the narrow distribution of population along the coastline. One interesting aspect of the impact of a nuclear detonation, single or multiple, on Bandar Abbas is its location on the coast of one of the most frequently traveled shipping lanes in the world. With a concerted planning effort with international participants, it would be feasible for the trauma and radiation victims to be transported across the Straits of Hormuz to nearby (and unaffected by the nuclear war as outside Iran) medical facilities in Oman and the United Arab Emirates. With appropriate triage of the victims to select transportable trauma victims, and with the inherent delay in the expression of toxicity in the radiation victims, it could be sufficiently productive to find, sort and transport these casualties to waiting hospital beds in these neighbors by air and sea transport.

The use of multiple 500 Kt weapons on the large city of Esfahan (Figure 14) would result in over 1.5 million fatalities (Table 4). This would result in about 140,000 and 24,000 3^rd^ and 2^nd^ degree burn victims there, respectively. About 9%, or 150,000 people, of the population would receive radiation exposures over 300 rem though there would be only a few hundred receiving this dose range from prompt radiation. In like manner, multiple 500 Kt detonations in Mashad (Figure 15) resulted in over 2 million fatalities, with a total casualty rate of 99.8%. This would include over 50,000 and 27,000 3^rd^ and 2^nd^degree burn casualties. In Shiraz, two 500 Kt weapons would result in 84% fatalities and a total of 95% casualties altogether (Figure 16). Tabriz with two 500 Kt has over 96% fatalities (Figure 17), while Ahvaz with a 500 Kt and a 250Kt has over 88% injuries (Figure 18). Of all the Iranian simulations, the multiple 250 Kt detonations in Kermanshah resulted in the largest number of prompt radiation victims, 17,570 (Figure 19). Kermanshah also had the highest number of trauma casualties resulting from >4.9 psi injuries. The striking aspect of multiple nuclear weapon strikes in compact Iranian cities is demonstrated by the combined fatalities and injuries for Kermanshah resulting in stunning 99.9% casualties!

### Stunning nuclear war casualty rates in Tehran

The high cost of nuclear war is seen most vividly in the consideration of multiple detonations of moderately sized nuclear weapons in the Iranian capital of Tehran, one of the most ancient urban areas in the world, whose long and celebrated history could be suddenly terminated in less than an hour. The concentration of over 8 million residents in Tehran would result in a large number of both fatalities and injuries, unlike the other simulations which are dominated primarily by fatalities (Figures 20, 21 and 22). In a comparison of the relative destructive power of increasing nuclear yields, it is seen that the percent of fatalities in Tehran steadily increase from 44% to 67% to 86% of the city’s population with the use of multiple 100 Kt, 250 Kt, and 500 Kt devices, respectively (Table 4). The percentage of injured residents of the capital, however, remain at about 20% of the population for 100 Kt and 250 Kt detonations, and actually decline by half for the 500 Kt strike.

No real appreciation of the magnitude of this disaster for Iran can be achieved without looking at the fatality numbers: 6 to 7 million deaths that would result from either five 250 or five 500 Kt weapons with the selected targeting. From the point of view of the unimaginable medical response challenge, there would be 1.5 million victims of thermal burns from either the 100 Kt or 250 Kt multiple weapon attacks, and from 750,000 to 880,000 severely (>300 rem) radiation-exposed patients. It is highly unlikely that the thermal burn victims that are immediately present after detonation will receive any care, and as the radiation victims begin presenting in the hours and days after the attack, they too are unlikely to be treated [33]. The 1.5 million trauma patients can be expected to occupy the full effort of whatever surviving medical response is available in the capital city area. Patients presenting with combined injuries including either thermal burns or radiation poisoning are unlikely to have favorable outcomes [30,31]. In the chaotic medical response of such a scenario, the best possible expectation is for a limited number of minor to moderate trauma victims to receive minimal care. Therefore, the already very high fatality rates could be expected to climb higher in the 24–72 hours immediately after a multiple nuclear weapon attack on Tehran.

### Nuclear war effects in Israel

Relative to the broad expanses of Iran, Israel is very small and narrow, with its population constrained primarily in a few urban areas. In the nuclear age of warfare, this makes the Israelis particularly vulnerable, lending strongly to the tension between the two nations. Assuming that Iran is only able to deliver five 15 Kt devices through Israeli defenses, detonations on three urban targets using four 15 Kt devices, and one strategic site in the Negev Nuclear Research Center are displayed. This is a reasonable assumption, as the rate of uranium enrichment to nuclear weapons grade in Iran is progressing rapidly enough to result in more than five weapons in the next few years. It is acknowledged that Iran could develop the weapon detonation technology during this same period. Also, missile development is proceeding briskly in Iran, and there is always the option of smuggling a weapon into Israel. It is this prospect that makes war between Israel and Iran more and more likely in the near term (i.e. next decade), as the balance of nuclear power will begin to move away from the speculated current dominance by Israel.

**Figure 23:**
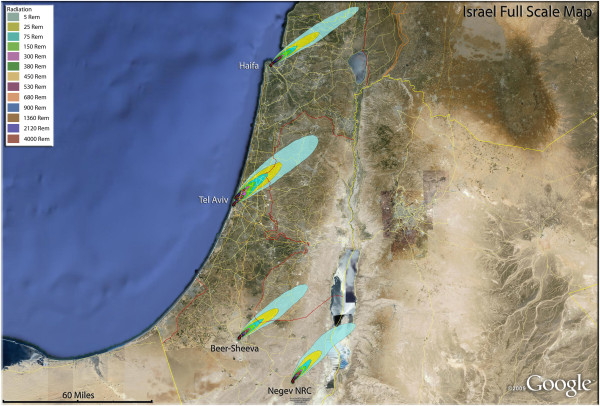
**Israel – Map of all modeled detonations.** Showing the radiation exposure plumes from all of the nuclear detonations nationwide for this scenario.

**Figure 24:**
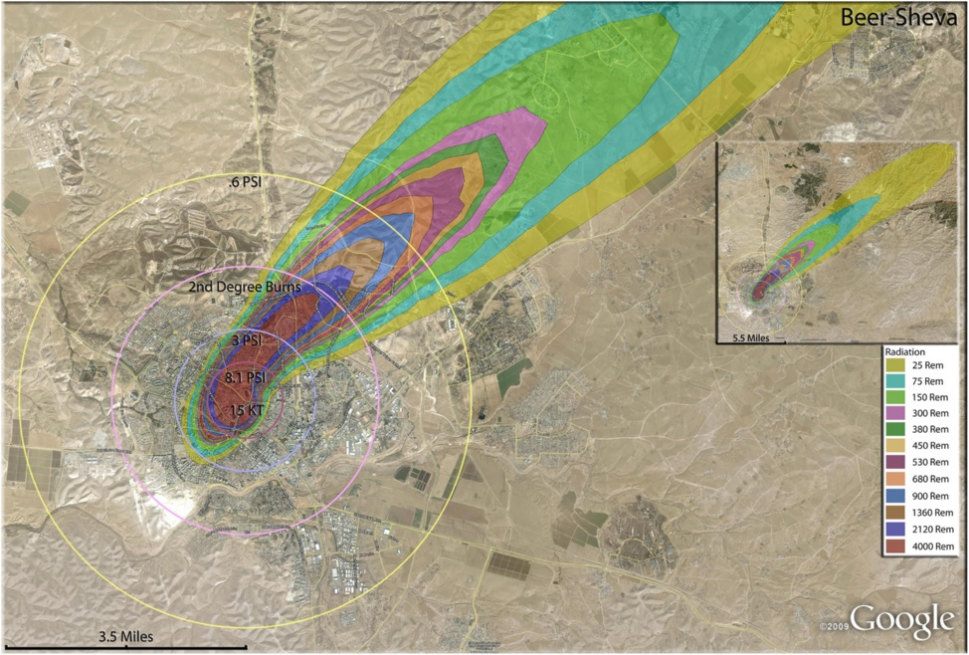
**Single 15 Kt nuclear weapon detonation casualties for Beer-Sheva, Israel.** Rings display trauma causalities for 8.1 and 3.0 psi, 2nd degree burns, and the outer ring is lower trauma casualties for 0.6 psi. Radiation exposures are delineated by color in the dispersion plume.

**Figure 25:**
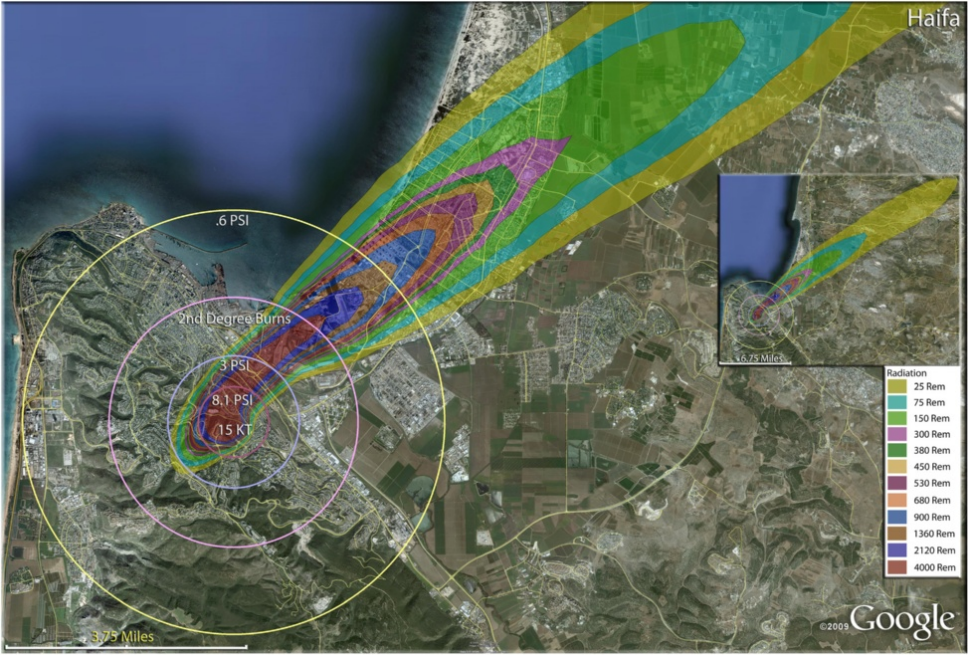
**Single 15 Kt nuclear weapon detonation casualties for Haifa, Israel.** Rings display trauma causalities for 8.1 and 3.0 psi, 2nd degree burns, and the outer ring is lower trauma casualties for 0.6 psi. Radiation exposures are delineated by color in the dispersion plume.

The devastating but relatively smaller casualty plumes (compared to Iran) from the Israeli targets are shown in Figure 23. Even these relatively small weapons result in significant destruction in the small confines of Israel. In the city of Beer-Sheva (Figure 24), half of the residents would be killed with a single weapon, with another sixth of the population being injured (Table 4). Nearly a fourth of the population would be in zones that would result in thermal injuries (if they survived the initial detonation). As in Iranian urban areas, the radiation plume primarily extends over uninhabited desert, largely negating radiation casualties (Figure 24). Similar fatality and injury ratios are seen in Haifa (Figure 25) (Table 4). Over 11,000 people would be radiation victims with serious radiation exposure (>300 rem), as the fallout plume extends along the Mediterranean coast. There would be over 40,000 trauma victims in Haifa, with the great majority of these in the larger 0.6 psi zone (Tables 5 and 6).

The location of Israel on the Mediterranean Sea allows for the possibility of the transport and even treatment of mass casualties onboard appropriately equipped vessels just off the coast. The proximity to European and American vessels that are virtually always in the Eastern Mediterranean means that especially with sufficient planning, these ships could be effectively utilized in the critical initial hours and days of a response to nuclear detonations in Israel (or in the surrounding countries, for that matter). Knowledge of the approximate location of trauma and burn victims, as depicted in this paper, could strategically direct the efforts of transport to selected vessels due to their location and relative ability to receive certain classes of patients. These same approaches could be made with aeromedical transport, which could be particularly useful for thermal burn victims, as they are transported to available hospital facilities in surrounding nations with which sufficient memoranda of understanding, emergency planning, and response supplies are positioned in advance.

**Figure 26:**
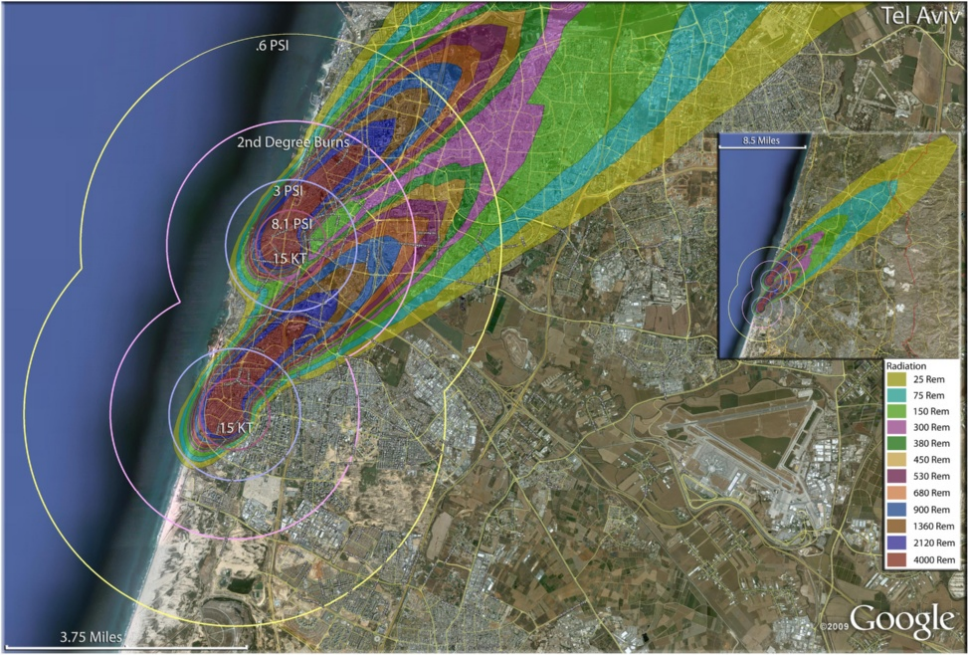
**Multiple detonation casualties for two 15 Kt nuclear weapons for Tel Aviv, Israel.** Rings display trauma causalities for 8.1 and 3.0 psi, 2nd degree burns, and the outer ring is lower trauma casualties for 0.6 psi. Radiation exposures are delineated by color in the dispersion plume.

In contrast to the 75-95% fatality outcomes for Iranian cities with the larger nuclear weapons, two 15 Kt devices used on Tel Aviv would result in 17% of the population being killed, though this still represents almost a quarter of a million people (Table 4). It is reasonable to assume that urban targeting in Tel Aviv would result in overlapping fields of burn, trauma and radiation victims (Figure 26). This multiple targeting, even with the relatively small 15 Kt devices, could result in over 100,000 people receiving thermal burn injuries. Another hundred thousand could receive potentially fatal doses of radiation from the two fallout plumes. Over 20,000 Israelis in Tel Aviv could receive serious trauma injuries resulting from >3psi exposure, while another 115,000 would be in the 0.6 psi zone, still capable of sustaining significant trauma injuries. As in other nuclear attack scenarios, this means that a number of these victims will sustain combination injuries [31-34]. Unlike most Iranian targets, the radiation plume extending from Tel Aviv would cover more populated areas in Israel, even reaching into populated areas of the West Bank.

## Conclusions

It has often been said that there are no winners in nuclear war, and that is certainly the case in the Middle East. The significant technological advantage of the Israeli nuclear forces (as they are assumed to exist) will give them a significant edge over the next decade, as Iran has the opportunity to enter nuclear war capability but will be far behind in number, yield, and delivery of nuclear weapons. However, the baseline destructive power of nuclear weapons will result in unacceptable losses in both nations in a nuclear war in the near future. In addition to the stunning human toll focused on in this publication, likely targeting of compact urban areas in both nations will result in devastating loss of critical industrial infrastructure and enormous economic decline. Based on recent large scale social disruptions in the expansive 20th Century wars, nuclear war in the Middle East will be likely to cause extremely damaging mental health and societal chaos on a focalized basis in these two nations as well as highly destabilizing ripple effects throughout the region and the rest of the world [25,27].

Health care delivery impacts from nuclear war in the Middle East will be dominated by the extremely high level of fatalities in Iranian cities, the inability to treat massive numbers of burn patients, huge logistical issues with trauma mass casualties, and relative unfamiliarity with radiation victim treatment. The lack of transportation and extensive water supplies has resulted in geographically compressed populations in Iranian cities that make them very vulnerable to high fatality rates from relatively large (>50 Kt) weapons. The fatal effects from blast, prompt radiation, and thermal burns essentially fills up the urban population zones in Iran, while the smaller weapon yields in Israel produce considerably smaller fatality rates there as large urban areas are still not yet reached.

As is the case in the simulation of nuclear attack on U.S. cities [11], the burn casualties in Iranian and Israeli cities tend to extend further out than the more serious trauma injuries, especially with the relatively larger nuclear weapons. This phenomenon will tend to produce large numbers of surviving thermal burn patients, many of them with concurrent trauma injuries, who will survive the nuclear detonation and will be in desperate need of medical care [30-32]. However, it is highly unlikely that these thousands of burn victims will receive any meaningful medical treatment, due to the high degree of effort currently necessary in emergency burn care (e.g. the high ratio of medical personnel to burn patients, and the need for sterile conditions) [1,32]. It is likely that the very limited surviving medical care will be apportioned to trauma care, where more robust outcomes can be achieved for each patient relative to available resources.

If any serious effort is to be made to meet the emergency medical requirements of the large number of widely dispersed thermal burn patients, new approaches will need to be developed. In order to reduce the number of burn patients subsequently lost to infection, it could be feasible to train portions of the populace (including but not limited to extended health care professionals not typically involved in emergency medical care) in wound debridement, and then make adequate burn medications accessible to them [1]. The large geographic distribution and number of burn victims, the scarcity of surviving traditional emergency medical personnel, and the lack of transport and accessible burn centers make such efforts indispensable. This is also problematic, though, in that criminal elements will want to gain access to the narcotic medications, and debridement is an excruciating experience under optimal conditions and much more so with minimally qualified personnel.

The very high casualty outcomes in nuclear war pose an extreme dilemma for those planning and executing medical response, which often or even usually results in despair and denial [30]. This can lead to a perception that such efforts at planning and response are not productive, such as in the use of prediction of casualty distributions as related in this publication. However, the many variations in nuclear war (as in all warfare) shows sufficient heterogeneity that allows for potentially effective changes in the strategy of the utilization of resources based on variations such as the approximate distribution of casualties. This is particularly the case with responding to the relatively smaller nuclear weapons such as the ones likely to be used by Iran on Israel in this simulation. While the trauma casualty numbers for the smaller nuclear detonations in Israel were still daunting, knowledge of the location of these trauma victims is highly useful in planning of patient transport, especially in an effective emergency response system as exists in Israel. For both large and relatively smaller nuclear weapons, predictions of the distribution of radiation casualties is essential to planning and response for the decontamination of these patients before transport, and for the prevention of contamination of rescue teams (i.e. planning of where to send the limited number of teams to both protect them and use them most efficiently). The location of accessible numbers of trauma victims from broken glass on the periphery of the blast zone is another example of a casualty distribution which is a favorable prediction for productive action for both smaller and larger nuclear weapons. In this manner, it can be a source of hope to be able to plan and respond in selected areas identified by predicted casualty distributions.

In the midst of such negative outcomes with thermal burn and trauma patients, it is interesting to note that the proportion of radiation victims in Middle Eastern urban nuclear attack is considerably less than that in similar simulations in the U.S [1]. The lack of urban sprawl and desert conditions in the Middle East results in very few people living outside of the immediate detonation areas where the thermal burn and trauma patients will be. A large proportion of the fatalities and injuries in the U.S. simulations were due to extended radiation plumes covering highly populated areas [11], which does not occur as readily in the Middle East, even with larger yield weapons. In the small confines of Israel, this lack of radiation victims was probably more related to the small size of the weapons likely to be available to Iran over the next decade.

As outlined for specific examples in Iran and Israel in the Results and Discussion, enlisting the mutual aid of other countries in responding to the very large medical response demands of nuclear war could be productive, especially with strategic planning and resource siting. In addition to the examples cited earlier for ship-mediated patient transport and treatment from the Israeli and Iranian coastlines, extensive use could be made with aeromedical transport. Such efforts would be greatly expedited with prior planning and mutual aid agreements, and in the actual event would be guided by estimates (verified over time) of casualty distributions and locations. The selection of sufficiently long airstrips could be made in close proximity to the estimated locations of the patient categories of interest, such as trauma victims on the periphery of the blast zone [1]. In this manner, it could be made sufficiently safe for the dispatch of rescue teams and the air transport itself (by avoiding radiation zones, traffic barriers, and security issues), and provide a better chance for getting certain patient categories to the air transport in time to actually help them. These considerations make a strong case for the political and diplomatic need for efforts to make these kinds of mutual aid arrangements in advance of nuclear war and other mass casualty surge planning in the Middle East.

The dramatic difference in fatality and injury numbers between Iran and Israel was predicated largely on the difference in numbers and explosive yields of the speculated arsenals of nuclear weapons in these nations over the next decade. There is a very significant difference in casualty rates between weapons in the range of 15 Kt and the larger weapons (>100 Kt) [11]. While casualty rates continue to go up with increasing yields beyond that, it is less dramatic and the differences between 250 Kt and 500 Kt detonations are not nearly as vivid. This shows the significant superiority of nuclear arsenals of advanced nuclear powers relative to nations in an earlier stage of development. Nations like Iran, North Korea, and Pakistan, which possess only 15 Kt devices, are at a disadvantage in a nuclear war with a well-developed nuclear power. In the case of the Middle East, the much smaller geographic makeup of Israel, which makes it vulnerable to nuclear attack, is greatly offset at this time by the assumption of vastly superior Israeli nuclear forces over the next decade [35]. By the end of that time, though, Iran can be expected to significantly close that gap as large scale production of nuclear weapons comes within its capability. It is exactly this scenario that makes a nuclear war in the Middle East in the near future more likely, with ominous consequences not only for Iran and Israel but the rest of the world.

## Competing interests

The authors declare that they have no competing interests.

## Authors’ contributions

CD was in charge of the research design/direction and drafted the manuscript; WB designed and conducted the nuclear war casualty simulation model, including methods text; DS conducted the GIS simulations; AC compiled the casualty distribution tables and figures; FB contributed to manuscript impact conclusions, limitations, and outcomes. All authors read and approved the final manuscript.

## References

[B1] Dallas CE Bell WC Prediction Modeling to Determine the Adequacy of Medical Response to Urban Nuclear Attack Disaster Med Public Health Prep 2007 1 2 80 89 10.1097/DMP.0b013e318159a9e3 18388634

[B2] Bell WC Dallas CE Vulnerability of populations and the urban health care systems to nuclear weapon attack – examples from four American cities Int J Health Geographics 2007 6 5 1 33 [http://www.ij-healthgeographics.com/content/6/1/5] 10.1186/1476-072X-6-5 PMC1828719 17328796

[B3] Cordesman AH Iran, Israel and Nuclear War 2007 Washington, D.C: Center for Strategic and International Studies (CSIS) [http://csis.org/program/burke-chair-strategy]

[B4] CIA Factbook 2009 Washington D.C [https://www.cia.gov/library/publications/the-world-factbook/geos/is.html]

[B5] Dolan P Capabilities of Nuclear Weapons, Defense Nuclear Agency Effects Manual Number 1 (EM-1) 1972 Washington, D.C: Defense Nuclear Agency

[B6] Oak Ridge National Laboratory’s LandScanTM 2007 Global Population Dataset Oak Ridge, TN [http://www.ornl.gov/sci/landscan/index.shtml]

[B7] ArcGis 9.3 ESRI Redlands, CA [http://www.esri.com/products#arcgis_panel]

[B8] Defense Threat Reduction Agency HPAC V4.04SP4 2005 Fort Belvoir, Va: DTRA

[B9] Glasstone S Dolan P Thermal Radiation and its effects 1977 The Effects of Nuclear Weapons: United States Department of Defense and the Energy Research and Development Administration

[B10] Eden L Whole World on Fire 2004 Ithaca, NY: Cornell University Press

[B11] Brode HL Small RD Soloman F, Marston RQ A review of the physics of large fires. The Medical Implications of Nuclear War 1986 Washington, DC: Institute of Medicine, National Academy Press

[B12] Binninger G Hodge JK Wright S Holl S Development of a fire prediction model for use within HPAC 2003 San Diego, CA: L3 Titan Corp

[B13] Craver RH Martin SB Bacon DP Doenges GR Samuels WB Nuclear Weapon Induced Urban Fires and Smoke Injection Technical Report Prepared for Director, Defense Supply Service, Belleview, Nebraska; 31 July, 1987 75

[B14] Diehl SR Keith JR Conley P Probabilistic thermal transmission curves. Fig 8.6 V1 Volume I – European and Mideast Results and Volume II-KSC Curve-fitted Scattered Radiation Database, DNA- TR-87-262-V1 and -V2) 1987

[B15] Office of Technology Assessment Effects of Nuclear Weapons 1979 Washington, DC 20402: Superintendent of Documents, U.S. Government Printing Office

[B16] Howe D National planning scenarios executive summaries 2005 Washington DC: The Homeland Security Council

[B17] Harney R Inaccurate Prediction of Nuclear Weapons Effects and Possible Adverse Influences on Nuclear Terrorism Preparedness Homeland Security Affairs 2009 5 Issue 3 [http://www.hsaj.org/?article=5.3.3]

[B18] WE Program Pittsburgh Defense Nuclear Agency 1997 Washington DC [http://nuclearweaponarchive.org/Library/Nukesims.html]

[B19] Postol TA Soloman F, Marston RQ Possible Fatalities from Superfires Following Nuclear Attacks in or near Urban Areas The Medical Implications of Nuclear Wa 1986 Washington DC: Institute of Medicine, National Academy Press

[B20] Daugherty WH Levi B von Hippel FN Soloman F, Marston RQ Casualties due to blast, heat, and radioactive fallout from various hypothetical nuclear attacks on the US The Medical Implications of Nuclear War 1986 Washington, DC: Institute of Medicine, National Academy Press

[B21] Anno GH Baum SJ Withers HR Young RW Symptomatology of acute radiation effects in humans after exposure to doses of 0.5-30Gy Health Physics 1989 56 6 821 838 10.1097/00004032-198906000-00001 2722506

[B22] Lawrence Livermore National Laboratory ES&H Manual Document 22.6 Exposure to Radiation in an Emergency 2003 Livermore, CA: LLNL

[B23] Bradley S Mazzola T Ross R Srinivasa D Verification and Validation of HPAC 3.0 Final Report on Contract DSWA-TR-98-66 1998 Dulles, VA 20166: Defense Threat Reduction Agency 269

[B24] The Department of Defense Verification, Validation and Accreditation (VV&A) Recommended Practices Guide DMSO, Chapter one of the VV&A Recommended Practices Guide 1996

[B25] The Hazard Prediction and Assessment Capability (HPAC) user’s guide version 4.0.3. Prepared for the Defense Threat Reduction Agency by Science Applications International Corporation, Rep. HPAC-UGUIDE-02-U-RAC0 2001 Fort Belvoir, VA: DTRA 602

[B26] DOD 5000.59. DOD Directive: Modeling and Simulation Management 2007 [http://www.dtic.mil/whs/directives/corres/pdf/500059p.pdf]

[B27] Binninger G Hodge J Wright S Holl S First uncertainty addendum to “Development of a Fire Prediction Model for use within HPAC” 2003 Fort Belvoir, VA: presented to Defense Threat Reduction Agency

[B28] Binninger G Hodge J Wright S Holl S Second uncertainty addendum to “Development of a Fire Prediction Model for use within HPAC” 2004 Fort Belvoir, VA: Section 7, Uncertainty, presented to Defense Threat Reduction Agency

[B29] Northrop J Handbook of Nuclear Weapons Effects, Effects Manual 1 1996 DSWA: Washington D.C

[B30] Abrams HL Von Kaenel WE Medical survivors of nuclear war: infection and the spread of communicable disease N Engl J Med 1981 305 1226 1232 10.1056/NEJM198111123052027 7290140

[B31] Abrams HL Medical resources after nuclear war: availability vs. need J Am Med Assoc 1984 252 653 658 10.1001/jama.1984.03350050041024 6737669

[B32] British Medical Association The medical effects of nuclear war 1983 London: BMA

[B33] Beker WK Buescher TM Cioffi WG Weiss JF, Brown D, Mac Vittie TJ Combined radiation and thermal injury after nuclear attack. Treatment of Radiation Injuries 1990 New York: Plenum

[B34] World Health Organization Effect of Nuclear War on Health and Health Services 1987 Geneva

[B35] Dallas CE Burkle FM Nuclear War in the Middle East: Where is the Voice of Medicine and Public Health? Prehospital Disaster Med 2011 26 05 383 385 10.1017/S1049023X11006613 22509536

